# Comparing Efficacy and Safety in Catheter Ablation Strategies for Paroxysmal Atrial Fibrillation: A Network Meta-Analysis of Randomized Controlled Trials

**DOI:** 10.3390/diagnostics12020433

**Published:** 2022-02-09

**Authors:** Emmanouil Charitakis, Silvia Metelli, Lars O. Karlsson, Antonios P. Antoniadis, Ioan Liuba, Henrik Almroth, Anders Hassel Jönsson, Jonas Schwieler, Skevos Sideris, Dimitrios Tsartsalis, Elena Dragioti, Nikolaos Fragakis, Anna Chaimani

**Affiliations:** 1 Department of Cardiology, Linköping University Hospital, 581 85 Linköping, Sweden; lars.o.karlsson@regionostergotland.se (L.O.K.); ioan.liuba@regionostergotland.se (I.L.); Henrik.almroth@regionostergotland.se (H.A.); anders.hassel.jonsson@regionostergotland.se (A.H.J.); 2 Research Center of Epidemiology and Statistics (CRESS-U1153), INSERM, Université de Paris, 75004 Paris, France; silvia.metelli@parisdescartes.fr (S.M.); anna.chaimani@inserm.fr (A.C.); 3 3rd Cardiology Department, Hippokrateion General Hospital, Aristotle University Medical School, 54124 Thessaloniki, Greece; aantoniadis@gmail.com (A.P.A.); fragakis.nikos@googlemail.com (N.F.); 4 Heart and Vascular Theme, Karolinska University Hospital, 171 76 Stockholm, Sweden; jonas.schwieler@sll.se; 5 Department of Cardiology, Hippokration Hospital, 11527 Athens, Greece; skevos1@otenet.gr; 6 Department of Clinical Physiology, Linköping University Hospital, 581 85 Linköping, Sweden; dtsartsalis@gmail.com; 7 Pain and Rehabilitation Centre, Linköping University Hospital, 581 85 Linköping, Sweden; elena.dragioti@liu.se; 8 Department of Health, Medicine and Caring Sciences, Linköping University, 581 83 Linköping, Sweden

**Keywords:** network meta-analysis, paroxysmal atrial fibrillation, catheter ablation, antiarrhythmic drugs

## Abstract

Although catheter ablation (CA) is an established treatment for paroxysmal atrial fibrillation (PAF), there is no consensus regarding the most efficient CA strategy. The objective of this network meta-analysis (NMA) was to compare the efficacy and safety of different CA strategies for PAF. A systematic search was performed in PubMed, Web of Science, and CENTRAL until the final search date, 5 October 2020. Randomised controlled trials (RCT) comparing different CA strategies and methods for pulmonary vein isolation (PVI) were included. Efficacy was defined as lack of arrhythmia recurrence after CA and safety as any reported complication related to the procedure during a minimum follow-up time of six months. In total, 43 RCTs comparing 11 different CA strategies involving 6701 patients were included. The risk of recurrence was significantly decreased in comparison with PVI with radiofrequency only for the following treatments: PVI with adjuvant ablation (RR: 0.79, CI: 0.65–0.97) and PVI with sympathetic modulation (RR: 0.64, CI: 0.46–0.88). However, PVI with radiofrequency was superior to non-PVI strategies (RR: 1.65, CI: 1.2–2.26). No statistically significant difference was found in safety between different CA strategies. Concerning different PVI strategies, no difference was observed either in efficacy or in safety between tested strategies. This NMA suggests that different PVI strategies are generally similar in terms of efficacy, while PVI with additional ablation or sympathetic modulation may be more effective than PVI alone. This study provides decision-makers with insights into the efficacy and safety of different CA strategies.

## 1. Introduction

Atrial fibrillation (AF) is the most common cardiac arrhythmia. In the U.S. alone, the prevalence of AF was estimated to be 5.2 million in 2010, while it is anticipated to double by 2030 [1]. AF constitutes a significant burden for patients and physicians [1], being one of the major causes of stroke, heart failure, and sudden death [2].

Patients experiencing short episodes of AF that terminate spontaneously or with intervention within seven days are classified as having paroxysmal AF (PAF) [2,3]. Catheter ablation (CA) and the use of antiarrhythmic drugs (AADs) are the most important heart rhythm (HR) control treatment options for these patients. These treatments have the potential to affect mortality and morbidity outcomes; however, the former has been difficult to prove [4]. Treatment with AADs has been questioned since it has been associated with side effects such as proarrhythmia; the latter hypothesised to explain that rhythm control through AADs does not offer mortality benefits [2]. However, a recent paper reported that early rhythm control therapy through AAD or CA was associated with a lower risk of adverse cardiovascular outcomes in patients with a short history of AF (≤1 year) [5]. Additionally, CA in a recent meta-analysis was associated with lower all-cause mortality and reduced recurrences of atrial arrhythmia compared with AADs [4].

Pulmonary vein isolation (PVI) has been the cornerstone of CA procedures for AF since the seminal observation of the initiation of AF by ectopic beats from the pulmonary veins [6,7]. CA procedures are differentiated by the energy source used, most frequently radiofrequency (RF) and cryothermal energy, as well as the strategy followed, such as PVI, non-PVI, or PVI with adjuvant ablation treatment. While CA is the mainstay HR control strategy in AF patients, the optimal CA strategy is under debate. Different treatment strategies have been suggested aiming at better long-term outcomes with regards to symptom and rhythm control, where many of them are less evaluated and different AF populations targeted. Prior meta-analyses on the topic have been conducted, but they were either inconclusive [8], focused on comparing CA to AADs [4], or compared different energy sources for the isolation of the pulmonary veins [9]. Thus, the need for a meta-analysis comparing the efficacy and safety of different CA strategies is emerging.

Network meta-analysis (NMA) is a statistical method that provides estimates of the relative effects for all available interventions, allowing comparison between every pair of treatments, even when they have not been compared directly in any trial [10,11]. NMA is an established method and has been applied in the field of cardiovascular medicine [12,13] and provides a powerful tool for the development of decision-making guidelines at different levels [14]. Thus, by employing the NMA methodology, we sought to compare the efficacy and safety of different CA strategies for the treatment of PAF.

## 2. Materials and Methods

### 2.1. Study Design

This NMA is reported following the Preferred Reporting Items for Systematic Reviews and Meta-analyses (PRISMA) extension statement for NMA [15] (Table S1 in Section S1 in Supplement). This NMA is based on previously published data; thus, it does not require ethical approval or consent to participate. The study protocol was registered in the prospective registry of systematic reviews, PROSPERO with registration number CRD42020169494, and has been published previously [16].

### 2.2. Eligibility Criteria and Type of Interventions

We included randomised controlled trials (RCTs), comparing different CA strategies for PAF in adults [2,17]. Studies that included patients with prior ablation (catheter, surgical or atrioventricular node ablation) or used a study design other than an RCT [16] were excluded.

The primary intervention of interest included CA strategies such as PVI, non-PVI approaches, PVI and adjuvant ablation treatments, PVI and sympathetic modulation, different PVI-strategies such as PVI RFA, PVI with cryoballoon ablation (CBA), laser-balloon ablation (LBA), or phase duty-cycled radiofrequency (PRF). All interventions are presented in Table 1 and Table S2 in Section S2 in the Supplement.

### 2.3. Search Strategy, Study, and Data Collection

The investigators (EC and DT) performed a comprehensive screening guided by the inclusion criteria to identify eligible studies (the search code is presented in Section S3, Supplement). We searched for relevant articles using PubMed, the Cochrane Central Register of Controlled Trials, and the Web of Science to the final search date, the 5 October 2020. Finally, references of previously published systematic reviews and included RCTs were screened for additional eligible studies.

The investigators (EC and DT) independently reviewed the titles and abstracts retrieved from the screening. For eligibility, inclusion criteria should be met, and the investigators should agree. Studies that were excluded (ineligible) were evaluated by both reviewers who agreed that they did not meet the inclusion criteria. In cases of disagreement, a third member was called in to adjudicate (ED). The full text of the remaining articles was obtained for eligibility control, and investigators (EC and DT) read each article and performed the data abstraction independently. Any disagreements were resolved by consultation with a third reviewer (ED) [16].

All data were summarised based on study characteristics (first author’s name, publication year, enrolment period, trial design), patient characteristics (age, sex, type of AF, background factors, etc.), intervention-related data (CA approach, fluoroscopy time, blanking period, follow-up time, methods used for the detection of possible recurrences, etc.), and outcome measures. The original authors were contacted for data requests when missing data was evident.

### 2.4. Outcomes

#### 2.4.1. Primary Outcomes

Efficacy outcome: Recurrence of AF or atrial tachycardia (AT) at ≥6 months after CA for AF in patients with PAF with a minimum duration of 30 s recorded on implantable loop recorder (ILR), pacemaker, defibrillator, ECG, or ambulatory ECG.

Safety: All reported complications related to the procedure (periprocedural or occurred during the follow-up).

#### 2.4.2. Secondary Outcomes

The secondary outcomes included:

Procedural time: defined as the time (measured in minutes) from the vascular access to the end of the procedure

All-cause mortality: from randomisation until the end of study follow-up.

### 2.5. Quality Assessment

The quality of the included RCTs was rated by the Cochrane Collaboration risk of bias (RoB) tool for RCTs (RoB V.2) [18]. RoB V.2 is structured into domains of bias, and a proposed decision about the RoB is generated based on answers to domain-relevant signalling questions. A decision can be of ‘low’ or ‘high’ RoB or raise ‘some concerns’ [18]. The way missing data were treated has been previously discussed [16] (more details are given in Section S4 in the Supplement).

### 2.6. Evaluation of Clinical Assumptions and Transitivity

Qualitative comparisons of study and population characteristics across trials were conducted to assess whether these were sufficiently homogeneous to be synthesised together. Transitivity is the fundamental assumption of NMA and refers to the validity of carrying out indirect comparisons between treatments via one or more intermediate comparators. This means that, for any three treatments A, B, and C, if direct evidence exists for A vs. B and A vs. C, we can indirectly get the effect of B vs. C via A as (effect of A vs. C)—(effect of A vs. B). We examined transitivity graphically by comparing the distribution of several clinical and demographical variables (such as age, sex, hypertension, structural heart disease (SHD), coronary artery disease (CAD), left atrial dimensions, etc.) that could act as effect modifiers across treatment comparisons.

### 2.7. Data Synthesis and Evaluation of Statistical Assumptions

For each comparison between at least two studies or more, we performed random-effects pairwise, and network meta-analysis (NMA) [19] using R software (R version 4.0.2, netmeta package, version 6.6-6) to estimate summary risk ratios for efficacy and safety (RRs), and summary mean differences (MDs) for procedural time (minutes). For ranking treatments in this NMA, we used a frequentist analogue to the surface under the cumulative ranking curve (SUCRA), the P-score.

In the NMA, we assumed a common heterogeneity parameter to be shared across comparisons within an outcome, and we estimated the treatment hierarchy for the primary outcomes using P-scores, which represent the average extent of certainty for each intervention to be better than the competing ones [20].

Statistical heterogeneity has been studied using visual inspection of the forest plots, along with the magnitude of the between-study variance ($\tau^{2}$) [21] while statistical inconsistency (i.e., the disagreement of direct and indirect evidence) was evaluated with: (a) the side-splitting method and (b) the design-by-treatment interaction model. The former assesses inconsistency for every comparison, while the latter provides a global test of the entire network.

### 2.8. Small-Study Effects and Additional Analyses

For each outcome, the presence of small-study effects was evaluated by comparison-adjusted funnel plots for all active strategies against control (PVI) [10,22]. Prespecified subgroup analyses were performed based on AF detection device, follow-up duration, and AAD or repeat ablation during follow-up. Possible sources of heterogeneity with regards to publication year were assessed through a post hoc subgroup analysis. If evidence of heterogeneity or inconsistency was found, metaregressions followed to investigate whether clinical demographical or other methodological characteristics were acting as effect modifiers. The impact of each covariate was assessed in independent univariate analyses, retaining only those variables for which data were available for at least 10 studies. Moreover, sensitivity analyses were performed for the primary outcomes, excluding studies with high RoB (as reported in the RoB section) and studies using nonirrigated RFA catheters. We also performed a sensitivity analysis, including studies involving antiarrhythmic drugs (AADs). Finally, evaluation of the overall credibility of the evidence was carried out through the CINeMA framework (https://cinema.ispm.Unibe.ch; last accessed: 2 December 2021) [23] (More details concerning the methodology of the trial can be found in Sections S5–S13 in the Supplement).

## 3. Results

### 3.1. Characteristics of the Included Studies and Risk of Bias Assessment

**Figure 1:**
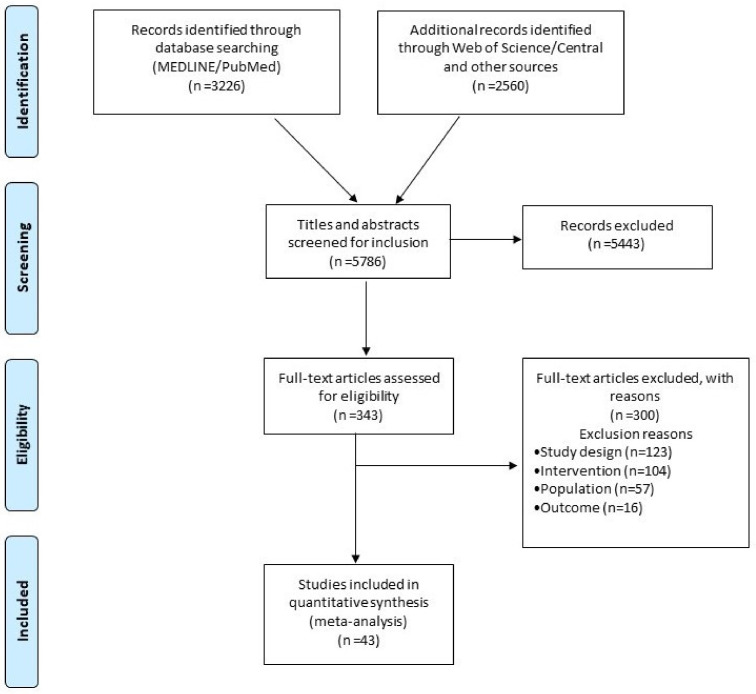
Prisma Flow chart diagram.

**Figure 2:**
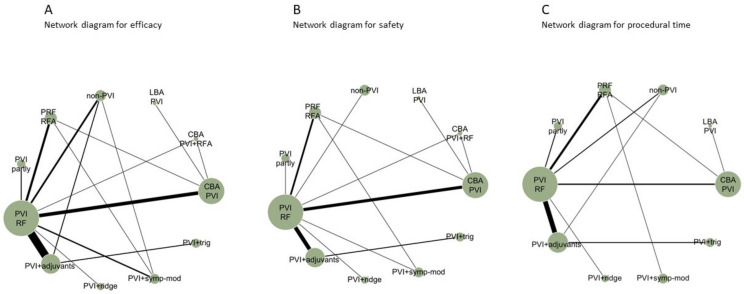
Network plots for efficacy (**A**), safety (**B**), and procedural time (**C**). Each treatment is represented as a node, and an edge is drawn between two nodes if direct evidence is available. The size of each node is proportional to the number of studies available in the corresponding comparison.

The literature search identified 5786 articles, of which 343 were considered eligible after the title and abstract review process. After a full-text review, 43 RCTs performed between 2003–2020 met the criteria for inclusion in the NMA (Figure 1), comparing 11 different CA strategies. Efficacy was reported by all studies, safety, and procedural time by 36 studies (84%) and 35 (81%) studies, respectively (Figure 2). Five RCTs had a high risk of bias (RoB V2 tool) (Section S5 in the Supplement). Deviations from the original protocol are presented in Section S6 in the Supplement.

### 3.2. Evaluation of Clinical Assumptions and Transitivity

Of the 6701 randomised patients, 69% were males, and the mean age was 58 years. The definition of effectiveness used in the included studies varied. This was due to different blanking periods and whether AADs or reablation were allowed according to the study protocol. We addressed this through sensitivity analyses. No important clinical differences in the distributions of most effect modifiers across different AF comparisons were observed for the majority of the characteristics analysed. Due to very limited available data for SHD and CAD, transitivity could not be properly evaluated concerning these two characteristics (Section S6 in the Supplement).

### 3.3. Relative Effects and Ranking of Treatments

#### 3.3.1. Primary Outcomes

**Figure 3:**
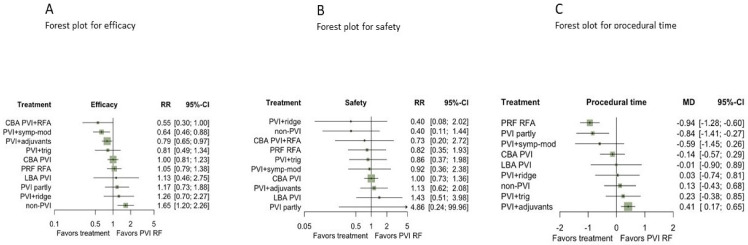
Forest plots for efficacy (**A**), safety (**B**), and procedural time (unit: minutes) (**C**) compared with PVI RF reporting the network meta-analysis RRs with their 95% CIs. Abbreviations: CBA: Cryoballoon ablation, HBA: Hot-balloon ablation, LBA: Laser-balloon ablation, PVI: Pulmonary vein isolation, RFA: radiofrequency ablation, trig: trigger ablation, PRF: Phase duty-cycled radiofrequency ablation.

**Figure 4:**
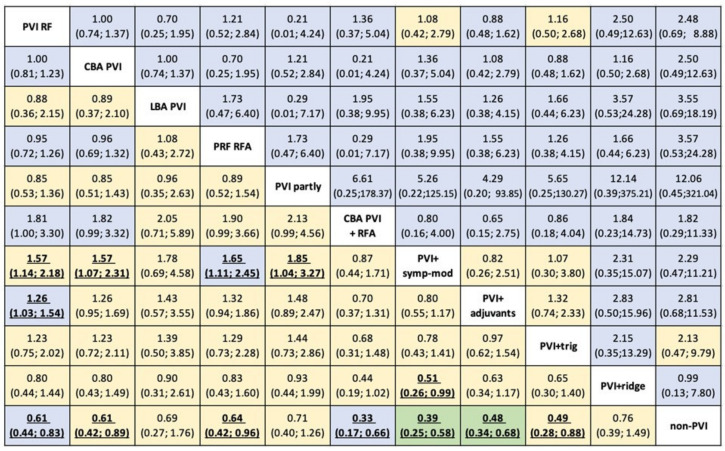
Risk ratios (RRs) for efficacy (lower triangle) and safety (upper triangle) with 95% CIs from network meta-analysis for 11 AF strategies available. Each cell is coloured by the certainty of evidence assessed for each comparison with CINeMA and classified as high (in green), moderate (in blue), low (in yellow). Abbreviations: CBA: cryoballoon ablation, HBA: hot balloon ablation, LBA: laser balloon ablation, PVI: pulmonary vein isolation, RFA: radiofrequency ablation, trig: trigger ablation, PRF: phase duty-cycled radiofrequency ablation.

Results from NMA evidence showed that non-PVI strategies (non-PVI) were statistically inferior to PVI with radiofrequency energy (PVI RF) (RR for non-PVI vs. PVI RF: 0.62, 95%CI: 0.44–0.89) (Figure 3, Figure 4 and Section S7 in the Supplement).

Strategies combining PVI with a) either adjuvant ablation (PVI+adjuvants) or b) sympathetic modulation (PVI+symp-mod) were significantly more efficient than PVI by RF as a stand-alone treatment (RR:0.79, 95%CI: 0.65–0.97, and RR: 0.64, 95%CI: 0.46–0.88), respectively (Figure 3A and Figure 4). Non-PVI strategies were statistically less efficacious than PVI RF. No significant difference was found between PVI RF and CBA PVI (RR:1, 95%CI: 0.81–1.23).

Regarding safety, no differences between CA strategies were evident from the NMA results (Figure 3B and Table S4 in Section S7 in the Supplement), nor a significant comparison between active treatments (Figure 4). Nonetheless, findings were quite imprecise for all comparisons, and thus no conclusive evidence could be supported.

According to the p-scores, PVI with adjuvants and PVI with symp-mod combinations of PVI appeared to be among the most effective treatments in patients with PAF (Section S7 in the Supplement), while the significant uncertainty in safety estimation hinders our confidence in conclusions about ranking for this outcome.

#### 3.3.2. Secondary Outcome

According to NMA results, PVI with adjuvant ablation was more time-consuming, compared with PVI RF (MD:0.41, 95%CI: 0.17–0.65 min), while PRF RFA [MD: −0.94, 95%CI: −1.28; −0.60 min] and partial PVI [MD = −0.84, 95%CI: −1.41; −0.27 min] were found to have shorter procedural time in comparison with PVI RF (Figure 3C). Moreover, none of the comparisons between active strategies performed better when contrasted with another active strategy (Table S5 in Section S7 in the Supplement).

All-cause mortality was not analysed due to the high prevalence of studies with zero events in both arms.

### 3.4. Assessment of Heterogeneity and Inconsistency

Network heterogeneity was low to moderate ($\tau^{2\;}$= 0.056 for efficacy, $\tau^{2}$ = 0 for safety, $\tau^{2}$ = 0.15 for procedural time). The overall design-by-treatment interaction test for inconsistency did not show any concerns of inconsistency for safety and procedural time but raised ‘some’ concern for efficacy (Section S8 in the Supplement). Similarly, the node-splitting method identified a few comparisons with ‘some’ concern of inconsistency for efficacy (Section S8, Supplement). However, in both models, this may be caused by a lack of statistical power in the tests since only a small number of studies per comparison inform the indirect estimation.

### 3.5. Small-Study Effects and Additional Analyses

From the graphical assessment of comparison-adjusted funnel plots, small-study effects did not appear to operate for any outcome. Even though the funnel plot of safety appears slightly less symmetrical, this might be attributable to the considerable imprecision observed in this outcome. Noteworthy, the procedural time funnel plot suggested the presence of a rather significant heterogeneity (Section S9 in the Supplement). This phenomenon can, to some extent, be explained by selective reporting.

Subgroup analyses showed favourable effects, i.e., more precise estimates (Section S10, Supplement) for some strategies; ECGs with longer recording times and not allowing AADs during follow-up. Conclusive evidence was not possible for follow-up time due to a large imbalance regarding group size. To inspect for potential causes of heterogeneity and inconsistency, we performed metaregression for efficacy. In metaregression, age and CAD were associated with a reduction in the heterogeneity of 14.7% and 30.7%, respectively, while the coefficients for all other variables were not significant and close to zero (Section S11 in the Supplement).

Sensitivity analyses excluding studies with a high risk of bias, or RCTs with non-irrigational RFA catheters, or including RCTs involving antiarrhythmic drugs (AAD) did not result in any significant risk change (Section S12 in the Supplement).

The sensitivity analysis including AADs included three additional RCTs (47 RCTs, *n* = 7633, 68% males, mean age 56 years). No significant change was found compared to the main analysis. Additionally, the majority of CA strategies, including PVI RF, CBA PVI, and PVI with adjuvants, were associated with a lower risk of recurrence when compared with AADs, RRs range from 0.22 (95%CI: 0.1,0.51) to 0.47 (95%CI: 0.23,0.98) (Section S12 in the Supplement).

### 3.6. Overall Quality of Evidence

According to the CINeMA evaluation (credibility of the evidence), confidence was mostly low for efficacy and moderate for safety. In both networks, the reasons for downgrading to moderate or low certainty were mainly related to the presence of significant concerns in imprecision, either alone or accompanied by some concerns in incoherence or indirectness. More details and concrete rules for downgrading are provided in Section S13 in the Supplement.

## 4. Discussion

Catheter ablation is an established treatment for the prevention of recurrences in patients with AF [2]. CA is generally recommended after failure of AADs, while recent guidelines [2] suggest that CA may be considered as first-line therapy in patients with PAF [2,24]. Regarding the recommended CA strategy for patients with PAF, PVI either in a point-by-point manner or as a one-shot device has been prioritised [2]. However, even if the results after PVI in patients with AF are satisfactory, SR in all PAF patients is not guaranteed [25,26]. This NMA explored and analysed existing data from 43 RCTs concerning 6701 patients to provide additional information about the most optimal CA strategy in patients with PAF.

The main findings of this NMA were:

- Based on the existing evidence, non-PVI strategies appear to be inferior in efficacy compared with the majority of PVI strategies.
- Different PVI strategies were found to be similarly effective.
- Different ablation strategies seem to be similar in terms of safety.
- Performing additional ablation to PVI is time-consuming, while there is no difference concerning procedural duration between the most used PVI strategies.
- All CA strategies except for non-PVI strategies and LBA appear to be superior to AADs.

### 4.1. Differences in Efficacy between Various CA Strategies

Pulmonary vein isolation is the strategy that gave a boost to CA for AF [2] and is the reason why CA has been established as the best option for HR control of AF. Non-PVI strategies have been tested against PVI in RCTs [27,28]. However, these studies are of small size compared with other CA trials [29] and include predominantly male participants. Thus, data from these studies may overestimate the efficacy of PVI. In the present study, we were able to confirm that CA strategies, including PVI, are superior in efficacy to non-PVI strategies.

Among the different methods used to achieve PVI, the first to be introduced was the use of radiofrequency applied in a point-by-point manner [6,30]. However, the complexity of RFA has limited the availability of CA to a few specialised centres, while the need for more simplified PVI techniques has been growing [29]. In recent years, single-shot devices for PVI have been introduced to meet this need, mainly in the form of balloon-based catheter ablation technologies. The CBA and the LBA systems are the main representatives of this category showing excellent efficacy and safety in some trials even when compared with RFA [29,31,32]. Our data, consistent with previous studies, did not detect strong evidence of superiority with regards to efficacy in one technology over the other [29,33,34]. However, a recent NMA by Gupta et al. [14] showed that PVI using RF combined with ablation index technology was superior in terms of efficacy compared with other options, such as CBA. This result is in contrast with our results. Nevertheless, we argue that the results of Gupta et al. should be treated with caution, as the authors included observational studies that raise the risk of bias, and the network was sparse.

Achieving long-term pulmonary vein isolation, either with a single shot device or a point-by-point manner, does not guarantee long-term freedom from atrial arrhythmia recurrence. In a study by Dukkipati et al., the reported AF recurrence rate one year after CA was 29% despite proven permanent PVI [35]. The reasons behind the lack of sustained response to PVI are not clear, but the existence of triggers outside the PV region has been shown to play an important role in some observational studies [36,37]. Hence, ablation of nonpulmonary vein triggers in specific regions such as the inferior mitral annulus, the interatrial septum, and the superior vena cava [25,38,39] can possibly add efficacy to PVI as a stand-alone therapy. Other CA strategies combined with PVI, such as posterior wall isolation (PWI), have shown promising results, especially in patients with persistent AF, but RCTs comparing PVI to PVI and PWI generated conflicting results [40]. Moreover, a subgroup analysis (Section S10, analysis 4) showed that the statistical superiority of PVI+adjuvant therapy compared with PVI RF is not supported by the RCTs with a publication year ≥ 2011.

Furthermore, autonomic modulation by, for example, GP as a complementary therapy to PVI, can improve CA’s efficacy, possibly by ablation of complex electrical activity areas [41]. Even though extensive wide area circumferential ablation of the pulmonary veins may have benefits beyond PVI, including concomitant ganglionated plexus modification, the use of a more dedicated strategy for the autonomic modulation, such as GP ablation or renal denervation, can benefit patients with PAF [26,41,42]. However, the lack of a sensitive and specific method for GP identification should be taken under consideration [7].

Taking these conclusions together, our study suggests that there is a place to consider additional ablation to the PVI strategy in patients with PAF. However, an individualised approach concerning the CA strategy is needed.

### 4.2. Safety

Even though complication rates regarding CA of AF are low, they remain a major concern for both patients and doctors. Accordingly, the aim of developing novel technologies in this field has been to improve the safety profile of CA as well as to enhance its efficacy.

The overall incidence of reported complications in this NMA was <6%, with a death rate of <1%. These complication rates are in line with previously published studies, including a worldwide survey [43,44]. Many RCTs included in this NMA reported a 0% mortality rate from randomisation to the end of follow-up. This was the reason why the secondary endpoint of all-cause mortality was unable to be processed.

The low incidence of complications observed in our NMA remained the same regardless of the CA strategy followed. This data may allow the electrophysiologist to focus on the best suitable method to enhance the efficacy of CA without being hindered by thoughts of taking an additional risk with a certain ablation strategy. However, it is crucial to acknowledge that most RCTs are performed by high volume academic centres, a fact that can lead to underestimation of the complication risk [43,45].

### 4.3. CA Strategies Compared with AADs

Early intervention in the course of AF by the implementation of AAD therapy or CA can limit its evolution by interrupting various pathophysiological changes [46,47]. Previous RCTs and meta-analyses are showing that arrhythmia recurrences were significantly less frequent in the case of specific CA strategies compared with AADs, especially in patients with PAF [4,32,48]. These data concur with the results from the sensitivity analysis, including AADs as a comparison arm presented in the present NMA. The only PVI strategy that failed to show superiority to AADs was LBA. This finding can be possibly explained by the lack of evidence when comparing LBA with AADs (no RCTs directly comparing LBA with AADs and only one RCT comparing LBA with CBA [31]).

### 4.4. Procedural Time

Single-shot PVI strategies were introduced in order to shorten procedural duration compared with point-by-point isolation of the pulmonary veins [29,49]. However, in our NMA, PVI by RFA and PVI with balloon devices did not differ in matters of procedural duration. This countertendency can be possibly explained due to different populations included and the evolution of RF techniques in the last five years.

As expected, the addition of adjuvant ablation therapy to PVI was more time-consuming compared with PVI as stand-alone.

### 4.5. Strengthens and Limitation

This NMA, comparing not only strategies for the PVI but all the available CA strategies for patients with PAF, is the first of its kind. It has the advantage of including 43 RCTs and more than 6701 patients providing the medical society with highly comprehensive results owing to the robustness of the statistical method used. The synthesis of the large number of included RCTs, performed in centres around the world, in combination with the opportunity of incorporating direct and indirect comparisons by the use of NMA, led to the interesting results previously discussed and enhanced the generalizability and applicability of our results to real-world scenarios.

However, our study, as every other meta-analysis, shares the limitations of the original studies. The existence of RCTs with a high risk of bias increases the risk of bias of the meta-analysis. Patients included in the original studies are assumed to have been sampled from the same theoretical pool of patients. Heterogeneity across studies is inevitable, as different methods are used across the original studies (i.e., for the detection of arrhythmia, blanking periods used, and the allowance of antiarrhythmic drugs during the follow-up varied). Additionally, the nature of the intervention can also impose heterogeneity as its efficacy may also depend on unmeasurable characteristics. In the case of the strategy arm of PVI with adjuvant therapies, many different extra ablation strategies have been included in combination with PVI, such as additional lines and superior vena cava isolation or posterior box isolation and electrocardiogram-based ablation. Thus, owing to the large heterogeneity, it is difficult to give a definite conclusion about this group’s superiority to PVI. Finally, it can be observed that the networks were quite sparse, in particular for safety and procedural time, making the results more difficult to interpret and the conclusions drawn more uncertain.

## 5. Conclusions

Overall, different PVI strategies were found to be similarly effective, while PVI with additional ablation therapy or autonomic modulation could increase the efficacy compared with PVI alone. Regarding CA safety, there was no observed difference between different strategies. CA of patients with PAF was statistically more efficient than AAD-therapy regardless of the CA strategy followed, except for non-PVI strategies and LBA. In summary, this NMA provides decision-makers with global and up-to-date evidence about the efficacy and safety of different CA strategies. The combination of these results and an individualised approach depending on patients’ needs and risk factors can lead to improved outcomes for PAF patients.

## Figures and Tables

**Table 1:** Interventions included in NMA (and their abbreviations).

Abbreviations of Interventions Included in NMA	Interventions Included in the NMA
CBA PVI	Pulmonary vein isolation with Cryoballoon ablation
CBA PVI and RFA	Pulmonary vein isolation with Cryoballoon ablation with adjuvant radiofrequency ablation
HBA PVI	Pulmonary vein isolation with hot balloon ablation
LBA PVI	Pulmonary vein isolation with laser balloon ablation
Non-PVI	Nonpulmonary vein isolation strategies such as ganglia plexi or electrocardiogram ablation
PVI RFA	Pulmonary vein isolation with radiofrequency ablation
PVI + sympathetic modulation	Pulmonary vein isolation with sympathetic modulation such as ganglia plexi ablation or renal denervation
PVI + adjuvants	Pulmonary vein isolation and adjuvant ablation such as additional lines or/and superior vena cava isolation or/and posterior box isolation or/and electrocardiogram-based ablation
PVI partly	Isolation of some pulmonary veins
PVI + ridge	Pulmonary vein isolation and ridge ablation
PVI + trig	Pulmonary vein isolation and trigger ablation
PRF RFA	Phase duty-cycled radiofrequency ablation for the isolation of the pulmonary veins

## Data Availability

Data are available in publicly accessible databases. The references of articles included are presented on the reference list and the background data in the Supplementary Material.

## Supplementary Materials

The following are available online at https://www.mdpi.com/article/10.3390/diagnostics12020433/s1. Sections S1–S13. References [16,18,25,26,27,28,29,31,33,34,39,41,42,48,50,51,52,53,54,55,56,57,58,59,60,61,62,63,64,65,66,67,68,69,70,71,72,73,74,75,76,77,78,79,80,81,82,83] are cited in the supplementary materials.
