# Supplementary material for: Comparing Efficacy and Safety in Catheter Ablation Strategies for Paroxysmal Atrial Fibrillation: A Network Meta-Analysis of Randomized Controlled Trials
Source: Diagnostics (Basel). 2022 Feb 9;12(2):433. doi: 10.3390/diagnostics12020433 (PMC8870912; doi:10.3390/diagnostics12020433)
Supplement: Supplementary file 1 [file diagnostics-12-00433-s001.zip › diagnostics-1526096-supplementary.pdf]

Supplementary content

Section S1. PRISMA NMA Checklist of Items to Include When Reporting A Systematic Review Involving a Network Meta-analysis

Section S2. Characteristics and list of RCTs included in the network meta-analysis

Section S3 Search Strategy

Section S4. Risk of bias assessment

Section S5. Deviations from the original protocol

Section S6. Evaluation of transitivity

Section S7. Additional results from pairwise and network meta-analysis

Section S8. Evaluation of inconsistency.

Section S9. Investigation of small-study effects.

Section S10. Subgroup analyses

Section S11. Meta-regression

Section S12. Sensitivity analyses

Section S13. Overall quality of the evidence with CINeMA assessment

References

Section S1. PRISMA NMA Checklist of Items to Include When Reporting A Systematic Review Involving a Network Meta-analysis

Table S1 PRISMA Checklist for network meta-analyses

Section/Topic	Item #	Checklist Item	Reported on Page #
**TITLE**			
Title	1	Identify the report as a systematic review *incorporating a network meta-analysis (or related form of meta-analysis).*	***1***
**ABSTRACT**			
Structured summary	2	Provide a structured summary including, as applicable:<br>**Background:** main objectives<br>**Methods:** data sources; study eligibility criteria, participants, and interventions; study appraisal; and *synthesis methods, such as network meta-analysis.*<br>**Results:** number of studies and participants identified; summary estimates with corresponding confidence/credible intervals; *treatment rankings may also be discussed. Authors may choose to summarize pairwise comparisons against a chosen treatment included in their analyses for brevity.*<br>**Discussion/Conclusions:** limitations; conclusions and implications of findings.<br>**Other:** primary source of funding; systematic review registration number with registry name.	**1**
**INTRODUCTION**			
Rationale	3	Describe the rationale for the review in the context of what is already known*, including mention of why a network meta-analysis has been conducted.*	***2***
Objectives	4	Provide an explicit statement of questions being addressed, with reference to participants, interventions, comparisons, outcomes, and study design (PICOS).	**2, ref 14**
**METHODS**			
Protocol and registration	5	Indicate whether a review protocol exists and if and where it can be accessed (e.g., Web address); and, if available, provide registration information, including registration number.	**2, 3 and ref 14**

Eligibility criteria	6	Specify study characteristics (e.g., PICOS, length of follow-up) and report characteristics (e.g., years considered, language, publication status) used as criteria for eligibility, giving rationale. *Clearly describe eligible treatments included in the treatment network, and note whether any have been clustered or merged into the same node (with justification).*	***2, 3***
Information sources	7	Describe all information sources (e.g., databases with dates of coverage, contact with study authors to identify additional studies) in the search and date last searched.	**2-4**
Search	8	Present full electronic search strategy for at least one database, including any limits used, such that it could be repeated.	**2-4 and ref 14**
Study selection	9	State the process for selecting studies (i.e., screening, eligibility, included in systematic review, and, if applicable, included in the meta-analysis).	**2-4 and ref 14**
Data collection process	10	Describe method of data extraction from reports (e.g., piloted forms, independently, in duplicate) and any processes for obtaining and confirming data from investigators.	**2-4**
Data items	11	List and define all variables for which data were sought (e.g., PICOS, funding sources) and any assumptions and simplifications made.	**2-4 and ref 14**
**Geometry of the network**	**S1**	Describe methods used to explore the geometry of the treatment network under study and potential biases related to it. This should include how the evidence base has been graphically summarized for presentation, and what characteristics were compiled and used to describe the evidence base to readers.	**4, 5, ref 14 and figure 2**
Risk of bias within individual studies	12	Describe methods used for assessing risk of bias of individual studies (including specification of whether this was done at the study or outcome level), and how this information is to be used in any data synthesis.	**4, Appendix 4**
Summary measures	13	State the principal summary measures (e.g., risk ratio, difference in means). *Also describe the use of additional summary measures assessed, such as treatment rankings and surface under the cumulative ranking curve (SUCRA) values, as well as modified approaches used to present summary findings from meta-analyses.*	**4, 5**
Planned methods of analysis	14	Describe the methods of handling data and combining results of studies for each network meta-analysis. This should include, but not be limited to:<br>• *Handling of multi-arm trials;*<br>• *Selection of variance structure;*<br>• *Selection of prior distributions in Bayesian analyses; and*<br>• *Assessment of model fit.*	**4, 5**
**Assessment of Inconsistency**	**S2**	Describe the statistical methods used to evaluate the agreement of direct and indirect evidence in the treatment network(s) studied. Describe efforts taken to address its presence when found.	**4, 5 Appendix 8**
Risk of bias across studies	15	Specify any assessment of risk of bias that may affect the cumulative evidence (e.g., publication bias, selective reporting within studies).	**4, 5 and ref 13**

Additional analyses	16	Describe methods of additional analyses if done, indicating which were pre-specified. This may include, but not be limited to, the following:<br>• Sensitivity or subgroup analyses.<br>• Meta-regression analyses;<br>• *Alternative formulations of the treatment network; and*<br>• *Use of alternative prior distributions for Bayesian analyses (if applicable).*	**4, 5**
**RESULTS†**			
Study selection	17	Give numbers of studies screened, assessed for eligibility, and included in the review, with reasons for exclusions at each stage, ideally with a flow diagram.	**9-10 and figure 1**
**Presentation of network structure**	**S3**	Provide a network graph of the included studies to enable visualization of the geometry of the treatment network.	**5 and Figure 2**
**Summary of network geometry**	**S4**	Provide a brief overview of characteristics of the treatment network. This may include commentary on the abundance of trials and randomized patients for the different interventions and pairwise comparisons in the network, gaps of evidence in the treatment network, and potential biases reflected by the network structure.	***5, 6***
Study characteristics	18	For each study, present characteristics for which data were extracted (e.g., study size, PICOS, follow-up period) and provide the citations.	**5, 6, Appendix4**
Risk of bias within studies	19	Present data on risk of bias of each study and, if available, any outcome level assessment.	**5, 6, Appendix 6**
Results of individual studies	20	For all outcomes considered (benefits or harms), present, for each study: 1) simple summary data for each intervention group, and 2) effect estimates and confidence intervals. *Modified approaches may be needed to deal with information from larger networks.*	***5, 6, Appendix 6,7***
Synthesis of results	21	Present results of each meta-analysis done, including confidence/credible intervals. *In larger networks, authors may focus on comparisons versus a particular comparator (e.g. placebo or standard care), with full findings presented in an appendix. League tables and forest plots may be considered to summarize pairwise comparisons.* If additional summary measures were explored (such as treatment rankings), these should also be presented.	***6, 7, figure 3, 4 and Appendix 7***

**Exploration for inconsistency**	**S5**	Describe results from investigations of inconsistency. This may include such information as measures of model fit to compare consistency and inconsistency models, *P* values from statistical tests, or summary of inconsistency estimates from different parts of the treatment network.	***6-8, Appendix 8***
Risk of bias across studies	22	Present results of any assessment of risk of bias across studies for the evidence base being studied.	**6-8, Appendix 4, 9, 10, 11, 12, 13**
Results of additional analyses	23	Give results of additional analyses, if done (e.g., sensitivity or subgroup analyses, meta-regression analyses*, alternative network geometries studied, alternative choice of prior distributions for Bayesian analyses,* and so forth).	***7-8, Appendix 10-12***
**DISCUSSION**			
Summary of evidence	24	Summarize the main findings, including the strength of evidence for each main outcome; consider their relevance to key groups (e.g., healthcare providers, users, and policy-makers).	**8-11**
Limitations	25	Discuss limitations at study and outcome level (e.g., risk of bias), and at review level (e.g., incomplete retrieval of identified research, reporting bias). *Comment on the validity of the assumptions, such as transitivity and consistency. Comment on any concerns regarding network geometry (e.g., avoidance of certain comparisons).*	**10-11**
Conclusions	26	Provide a general interpretation of the results in the context of other evidence, and implications for future research.	**11**
**FUNDING**			
Funding	27	Describe sources of funding for the systematic review and other support (e.g., supply of data); role of funders for the systematic review. This should also include information regarding whether funding has been received from manufacturers of treatments in the network and/or whether some of the authors are content experts with professional conflicts of interest that could affect use of treatments in the network.	***11***

Section S2. Characteristics and list of RCTs included in the network meta-analysis

Table S2. Characteristics of the 43 RCTs included in the network meta-analysis.

Study	Year of publication	blanking period (weeks)	follow up period (months)	total number of patients	Strategies	Number of patients	Age (mean ± SD)	Sex (% male)	Hypertension (%)	CAD (%)	CHF (%)	SHD (%)	LVEF % (mean±SD)	LAD mm(mean±SD)
Oral et al. (50)	2003	4	6	80	PVI RFA	40	51±10	31 (78)	NR	NR	NR	NR	55±4	40±5
					PVI+adjuvant	40	54±11	31 (78)	NR	NR	NR	NR	57±5	41±6
Katritsis et al. (51)	2004	NR	12	52	PVI partly	27	54±9	22 (82)	15 (54)	3 (11)	NR	NR	NR	NR
					PVI RFA	25	50±10	21 (84)	12 (48)	4 (16)	NR	NR	NR	NR
Hocini et al. (52)	2005	0	12	90	PVI+adjuvant	45	54±10	37 (82)	NR	NR	NR	10 (22)	67±8	54±8
					PVI RFA	45	55±8	34 (76)	NR	NR	NR	15 (33)	67±11	51±8
Liu et al. (53)	2006	12	9	110	PVI+adjuvant	55	58±8	35 (64)	NR	NR	NR	NR	63±6	37±4
					PVI RFA	55	57±10	38 (69)	NR	NR	NR	NR	64±7	38±4
Wang et al. (54)	2008	4	12	106	PVI+adjuvant	54	65+9	30 (58)	12 (23)	4 (8)	NR	NR	62+5	37+3
					PVI RFA	52	67+9	28 (52)	10 (19)	3 (6)	NR	NR	62+4	37+3
Deisenhofer et al. (55)	2009	0	6	98	PVI+adjuvant	48	55±10	41 (82)	NR	NR	NR	34 (68)	NR	44 ±5
					PVI RFA	46	58±10	33 (69)	NR	NR	NR	28 (58)	NR	43 ±6
Di Biase et al. (27)	2009	8	12	103	PVI+adjuvant	34	58±8	30 (88)	12 (35)	NR	NR	NR	54±6	41±5
					PVI RFA	35	57±8	29 (83)	12 (34)	NR	NR	NR	55±8	43±6
					non-PVI	34	60±9	26 (76)	13 (38)	NR	NR	NR	56±6	41±5
Sawhney et al. (56)	2010	12	12	66	PVI+adjuvant	33	59±10	25 (76)	NR	NR	NR	NR	61±4	37±4
					PVI RFA	33	55±12	23 (70)	NR	NR	NR	NR	62±6	36±3

Chen et al. (28)	2011	12	12	118	PVI+adjuvant	58	56 ±11	40 (68)	12 (20.6)	2 (3)	NR	NR	65±3	34±4
					PVI RFA	35	52±13	39 (67)	5 (20.8)	1 (5)	NR	NR	66±4	35±4
					non-PVI	24	58±9	23 (66)	10 (29)	3 (9)	NR	NR	66±5	36±4
Katritsis et al. (57)	2011	12	12	160	PVI+sympathetic modulation	34	55±12	25 (74)	16 (47)	NR	NR	NR	56±7	42±5
					PVI PRF	33	53±11	26 (79)	20 (61)	NR	NR	NR	56±5	41±3
Gavin et al. (58)	2012	12	18		PVI+adjuvant	20	67±14	15 (75)	8 (40)	NR	NR	NR	64.8±12	41±10
					PVI RFA	22	68±12	15 (68)	8 (36)	NR	NR	NR	64±14	41±9
Lin et al. (59)	2012	12	12		PVI+adjuvant	63	56 ± 9	42 (66)	18 (29)	NR	17 (27)	NR	61 ± 6	39 ± 5
					PVI+triggers	63	53 ± 11	40 (64)	10 (15)	NR	11 (18)	NR	61 ± 8	38 ± 7
Mun et al. (60)	2012	12	15.6		PVI+adjuvant	45	54±11	41 (79)	22 (42)	NR	1 (2)	NR	64±7	41±5
					PVI RFA	46	55±13	37 (71)	21 (40)	NR	1 (2)	NR	65±6	39±5
					PVI+adjuvant	43	59±11	41 (79)	24 (46)	NR	1 (2)	NR	64±8	40±4
Katritsis et al. (41)	2013	12	24	242	PVI+sympathetic modulation	82	56±9	57 (70)	58 (71)	NR	NR	NR	62±8	48±6
					PVI RFA	78	56±8	53 (68)	63 (81)	NR	NR	NR	63±7	48±7
					non-PVI	82	56±8	49 (60)	63 (77)	NR	NR	NR	63±7	49±6
Fichtner et al. (61)	2013	NR	12	207	PVI partly	105	59±12	74 (70)	69 (66)	11 (10.4)	NR	NR	NR	44±6
					PVI RFA	102	61±9	72 (71)	57 (56)	15 (15)	NR	NR	NR	44±6
Atienza et al. (62)	2014	8	12	232	PVI+adjuvant	54	54±12	40 (73)	24 (44)	NR	NR	12 (22)	60±9	40±6
					PVI RFA	58	53±10	49 (84)	17 (29)	NR	NR	7 (12)	60±9	40±5
Kang et al. (63)	2014	12	12	200	PVI+adjuvant	100	58+12	75 (75)	53 (53)	NR	NR	3 (3)	65+9	40+6
					PVI RFA	100	56+12	74 (100)	41 (41)	NR	NR	3 (3)	63+10	40+6
Nuhrich et al. (64)	2014	12	12	68	PVI+adjuvant	35	63+2	56 (63)	20 (57)	3 (9)	0	NR	68+1	40+1
					PVI RFA	33	59+2	20 (61)	17 (52)	2 (6)	0	NR	67+1	40+1
Arbelo et al. (65)	2014	12	12	120	PVI+adjuvant	59	55±11	42 (71)	19 (32)	4 (7)	NR	14 (24)	62±7	41±6

					PVI RFA	59	55±12	42 (69)	24 (39)	2 (3)	NR	7 (12)	62±5	41±6
Faustino et al. (66)	2015	12	12	150	PVI+adjuvant	75	62±9	48 (64)	48 (64)	5 (6.7)	NR	NR	59±7	44±3
					PVI RFA	75	63±8	44 (59)	56 (75)	9 (12.0)	NR	NR	59±7	44±3
Da Costa et al. (67)	2015	8	15	100	PVI+adjuvant	51	55±10	40 (78)	16 (31)	NR	NR	8 (16)	63±7	42±7
					PVI RFA	49	58±9	39 (80)	16 (33)	NR	NR	16 (32)	64±7	39±6
Kim et al. (68)	2015	12	12	100	PVI+adjuvant	50	58+11	35 (70)	22 (44)	NR	1 (2)	NR	65+7	41+7
					PVI RFA	50	55+12	40 (80)	16 (32)	NR	2 (4)	NR	64+8	39+6
Kiuchi et al. (26)	2018	12	12	69	PVI+sympathetic modulation	33	57±7	25 (76)	33 (100)	5 (15)	NR	NR	62±7	NR
					PVI RFA	36	58±5	30 (83)	36 (100)	9 (25)	NR	NR	61±6	NR
Steinberg et al. (42)	2019	12	12	302	PVI+sympathetic modulation	147	59 (54-65)	91 (59)	154 (100)	14 (9)	NR	NR	62±5	48±3
					PVI RFA	141	60 (58-65)	91 (62)	148(100)	10 (7)	NR	NR	62±5	47±3
Lee et al. (25)	2018	12	12	500	PVI+triggers	229	56±11	186 (74)	111 (44)	NR	10 (4)	NR	57±3	39±5
					PVI+adjuvant	240	56±11	187 (75)	97 (39)	NR	12 (5)	NR	56±4	39±6
Gaita et al. (69)	2008	8	12	125	PVI+adjuvant	84	56±10	NR	NR	NR	NR	NR	NR	44±6
					PVI RFA	41	53±9	NR	NR	NR	NR	NR	NR	42±7
Bulava et al. (70)	2010	4	6	102	PRF PVI	51	57± 10	36 (71)	15 (29)	2 (4)	NR	NR	70±6	41±5
					PVI RFA	51	60±12	30 (60)	18 (35)	3 (3)	NR	NR	68±8	40±4
Corrado et al. (39)	2010	8	12	134	PVI+adjuvant	61	NR	NR	NR	NR	NR	NR	NR	NR
					PVI RFA	73	NR	NR	NR	NR	NR	NR	NR	NR
Bordignon et al. (31)	2013	12	12	140	LBA PVI	68	63±9	43 (61)	42 (60)	9 (13)	NR	8 (11)	63±6	40±5
					PVI RFA	65	63±12	49 (70)	44 (63)	4 (6)	NR	4 (5)	63±4	40±4
McCready et al. (71)	2014	12	12	186	PRF PVI	92	58±12	58 (63)	26 (28)	NR	NR	6 (7)	64±6	38±7
					PVI RFA	91	62±11	58 (64)	22 (24)	NR	NR	3 (3)	62±11	39±5
Perez-Castellano et al. (34)	2014	12	12	50	CBA PVI	25	56 (40–61)	17 (68)	6 (24)	NR	NR	4 (16)	NR	NR
					PVI RFA	25	58 (45–62)	22 (88)	8 (32)	NR	NR	4 (16)	NR	NR

Hunter et al. (72)	2015	12	12	237	CBA PVI+RFA	79	58±12	49 (63)	30 (38)	5 (6)	9 (12)	NR	NR	43±4
					PVI RFA	77	61±12	47 (61)	23 (30)	6 (8)	4 (5)	NR	NR	43±5
					CBA PVI	78	56±11	56 (72)	27 (35)	6 (8)	7 (9)	NR	NR	42±4
McLellan et al. (73)	2015	12	12	334	PVI and ridge	117	59±10	79 (68)	45 (38)	9 (8)	4 (4)	NR	60±7	NR
					PVI RFA	117	59±9	75 (64)	44 (38)	13 (11)	1 (1)	NR	59±7	NR
Luik et al. (33)	2015	12	12	322	CBA PVI	144	61 (54, 66)	100 (64)	96 (62)	19 (14)	NR	NR	NR	NR
					PVI RFA	147	60 (54, 67)	91 (57)	103 (66)	20 (14)	NR	NR	NR	NR
Podd et al. (74)	2015	12	12	50	PRF PVI	25	68±10	9 (36)	12 (48)	1 (4)	NR	NR	60±8	37±8
					PVI RFA	25	67±13	13 (52)	9 (36)	2 (8)	NR	NR	62±5	40±6
Boersma et al. (75)	2016	12	12	120	PRF PVI	59	56±9	48 (79)	NR	3 (5)	NR	NR	NR	41±6
					PVI RFA	58	57±9	42 (72)	NR	5(8)	NR	NR	NR	40±6
Kuck et al. (29)	2016	12	18	762	CBA PVI	374	60±10	221 (59)	215 (58)	31 (8)	111 (29)	NR	NR	41±7
					PVI RFA	376	60±9	236 (63)	221 (59)	31 (8)	98 (26)	NR	NR	41±6
Schirdewan et al. (76)	2017	12	12	44	PRF PVI	15	63 (56–68)	12 (80)	9 (43)	3 (14.3)	1(4.8)	NR	NR	NR
					CBA PVI	21	62 (53–68)	13 (61)	15 (65)	7 (30.4)	0	NR	NR	NR
Davtyan et al. (77)	2018	12	12	89	CBA PVI	45	58±8	22 (49)	35 (78)	4 (9)	NR	NR	NR	41±3
					PVI RFA	44	56±12	19 (43)	34 (77)	2 (5)	NR	NR	NR	40±4
Kircher et al. (78)	2018	12	12	60	PVI+adjuvant	35	NR	NR	NR	NR	NR	NR	NR	NR
					PVI RFA	25	NR	NR	NR	NR	NR	NR	NR	NR
You et al. (79)	2019	NR	12	210	CBA PVI	140	60	81 (58)	85 (61)	1 (1)	10 (7)	NR	NR	36±4
					PVI RFA	70	58±10	41 (59)	35 (54)	2 (3)	5 (7)	NR	NR	36±5
Giannopoulos et al. (80)	2019	8	6	120	CBA PVI	80	61 (55-67)	NR	41 (51)	6 (8)	2 (3)	NR	60 IQR(54-65)	40 IQR(36-43)
					PVI RFA	40	58 (53-66)	NR	18 (45)	2 (5)	2 (5.0)	NR	60 IQR(55-65)	42 IQR(38-44)
Andrade et al. (81)	2019	12	12	346	CBA PVI	120	60±10	152 (61)	80 (34)	19 (8)	4 (2)	NR	59±5	38±10
					PVI RFA	62	59±9	79 (69)	40 (35)	6 (5)	2 (2)	NR	59±7	37±9

Section S3 SEARCH STRATEGY

Pubmed

("catheter ablation" OR RFA OR "radiofrequency ablation" OR ablation OR CA OR cryoablation OR cryoballoon OR pulmonary vein isolation) AND ("auricular fibrillation" OR "atrial fibrillation") AND ("Clinical Trial" [PT:NoExp] OR "clinical trial, phase i"[pt] OR "clinical trial, phase ii"[pt] OR "clinical trial, phase iii"[pt] OR "clinical trial, phase iv"[pt] OR "controlled clinical trial"[pt] OR "multicenter study"[pt] OR "randomized controlled trial"[pt] OR "Clinical Trials as Topic"[mesh:noexp] OR "clinical trials, phase i as topic"[MeSH Terms:noexp] OR "clinical trials, phase ii as topic"[MeSH Terms:noexp] OR "clinical trials, phase iii as topic"[MeSH Terms:noexp] OR "clinical trials, phase iv as topic"[MeSH Terms:noexp] OR "controlled clinical trials as topic"[MeSH Terms:noexp] OR "randomized controlled trials as topic"[MeSH Terms:noexp] OR "early termination of clinical trials"[MeSH Terms:noexp] OR "multicenter studies as topic"[MeSH Terms:noexp] OR “Double-Blind Method”[Mesh] OR ((randomised[TIAB] OR randomized[TIAB]) AND (trial[TIAB] OR trials[tiab])) OR ((single[TIAB] OR double[TIAB] OR doubled[TIAB] OR triple[TIAB] OR tripled[TIAB] OR treble[TIAB] OR treble[TIAB]) AND (blind*[TIAB] OR mask*[TIAB])) OR ("4 arm"[tiab] OR "four arm"[tiab]))

Cochrane central database of clinical trials

(¨atrial fibrillation¨ OR ¨auricular fibrillation¨ OR ¨AF¨) AND (¨radiofrequency ablation¨ OR ¨catheter ablation¨ OR ¨ablation¨ OR ¨CA¨ OR ¨cryoablation¨ OR ¨cryoballoon¨ OR ¨pulmonary vein isolation¨)

Web of science

(TS=(atrial fibrillation) AND TS=(ablation OR CA OR cryoablation OR cryoballoon OR pulmonary vein isolation) AND TS=(random*)) AND LANGUAGE: (English) AND DOCUMENT TYPES: (Article)

Section S4. RISK OF BIAS ASSESSMENT

The Cochrane Collaboration RoB tool for randomized trials (RoB V.2) was used to rate the quality of the included RCTs. RoB V.2 is structured into domains of bias, within each domain, a series of 'signaling questions' aim to obtain information about features of the trial that are relevant. A proposed decision about the RoB is generated based on answers to the signaling questions. A decision can be of 'low' or 'high' RoB or rise 'some concerns (18).

The way missing data were treated has been previously described (16). In short, in case of missing data the original authors have been contacted. Missing outcome data have been assessed with the use of RoB.

If a study was deemed to be at 'high risk' of bias in at least one domain or to have 'some concerns' for multiple domains, the overall risk of bias of the particular study has been judged to be of 'high risk' of bias. If a study was judged to have 'some concerns' in at least one domain for the result, then the overall risk of bias of the study should have also raised some concerns. Finally, if all the domains were of low risk of bias, then the overall judgment of the study would also have been of 'low risk' of bias.

It must be noted that the included RCTs in this NMA concern invasive procedures in which blinding of the operators was not possible. However, blinding of patients was still feasible.

Study	Risk of bias domains					
	D1	D2	D3	D4	D5	Overall
Andrade et al.	+	-	+	+	+	-
Arbelo et al.	+	-	-	+	+	-
Atienza et al.	+	-	+	+	+	-
Boersma et al.	+	-	+	+	+	-
Bordignon et al.	-	-	+	+	+	-
Bulava et al.	-	-	+	+	+	-
Chen et al.	-	X	+	+	+	X
Corrado et al.	+	X	+	+	+	X
Da Costa et al.	-	-	+	+	+	-
Davtyan et al.	-	-	+	+	-	X
Deisenhofer et al.	+	-	X	+	+	X
Di Biase et al.	+	-	+	+	+	-
Faustino et al.	+	-	+	+	+	-
Fichtner et al.	-	-	+	+	+	-
Gaita et al.	-	-	+	+	+	-
Gavin et al.	-	-	+	+	+	-
Giannopoulos et al.	-	-	+	+	+	-
Hocini et al.	-	-	+	+	+	-
Hunter et al.	+	-	+	+	+	-
McCready et al.	+	-	+	+	+	-
Kang et al.	-	-	+	+	+	-
Katritsis et al.a	-	-	+	+	+	-
Katritsis et al.b	-	-	+	+	+	-
Katritsis et al.c	+	-	+	+	+	-

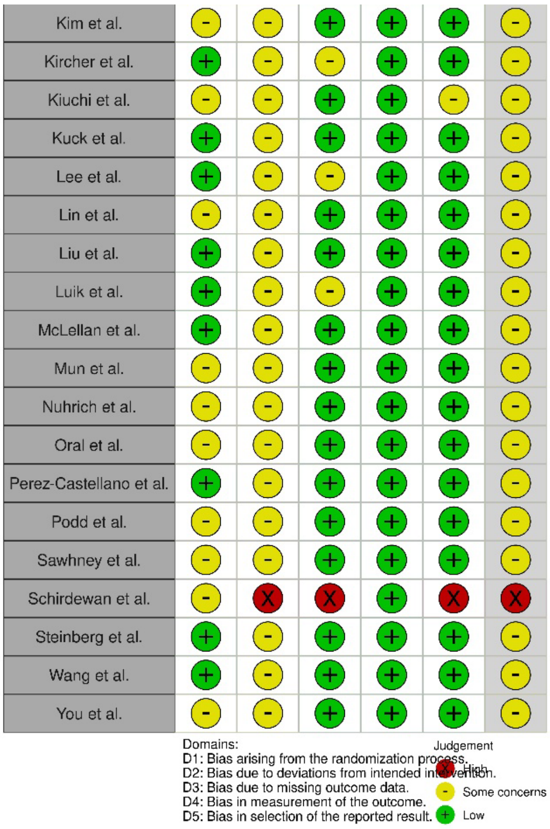
Kim et al.
Kircher et al.
Kiuchi et al.
Kuck et al.
Lee et al.
Lin et al.
Liu et al.
Luik et al.
McLellan et al.
Mun et al.
Nuhrich et al.
Oral et al.
Perez-Castellano et al.
Podd et al.
Sawhney et al.
Schirdewan et al.
Steinberg et al.
Wang et al.
You et al.
Domains:
D1: Bias arising from the randomization process.
D2: Bias due to deviations from intended intervention.
D3: Bias due to missing outcome data.
D4: Bias in measurement of the outcome.
D5: Bias in selection of the reported result.
Judgement
High
Some concerns
Low

Section S5. DEVIATIONS FROM THE ORIGINAL PROTOCOL

The endpoint of all-cause mortality was not analyzed due to the high prevalence of studies with zero events in both arms (accounting for more than 92% of the reported measures).

Studies including AADs as a comparing arm were excluded from the main analysis. This was decided mainly due to transitivity issues. It seemed from the transitivity investigation (Appendix 6) that the distributions of the main effect modifiers were not the same between studies which included AADs and those with only CA-arms. Studies comparing AADs with CA were included in a sensitivity analysis.

We also experienced problems regarding consistency in our original analysis (statistical disagreement between direct and indirect evidence in the network). To achieve a larger number of studies per comparison and improve consistency, we categorize CA-strategies in larger groups (table 1) compared to those presented in the published protocol (16). Hence the planned sensitivity analysis excluding the comparing arm of PVI with renal denervation (RDN) was not feasible.

We performed also a post-hoc subgroup-analysis in relation to publication year to reveal possible sources of heterogeneity depending on if the data were more recent.

Section S6. EVALUATION OF TRANSITIVITY

In network meta-analysis, transitivity refers to the fact that the relative effect between treatment A and treatment B can be validly compared indirectly through one or more intermediate comparators. To evaluate this, we report boxplots for the distributions of seven potential effect modifiers: mean age, percentage of males, presence of hypertension, coronary artery disease (CAD), structural heart disease (SHD), left atrial dimensions and left ventricle (LV) ejection fraction (EF) across the available direct comparisons. If effect modifiers are similarly distributed across comparisons, the transitivity assumption is considered to hold in the network.

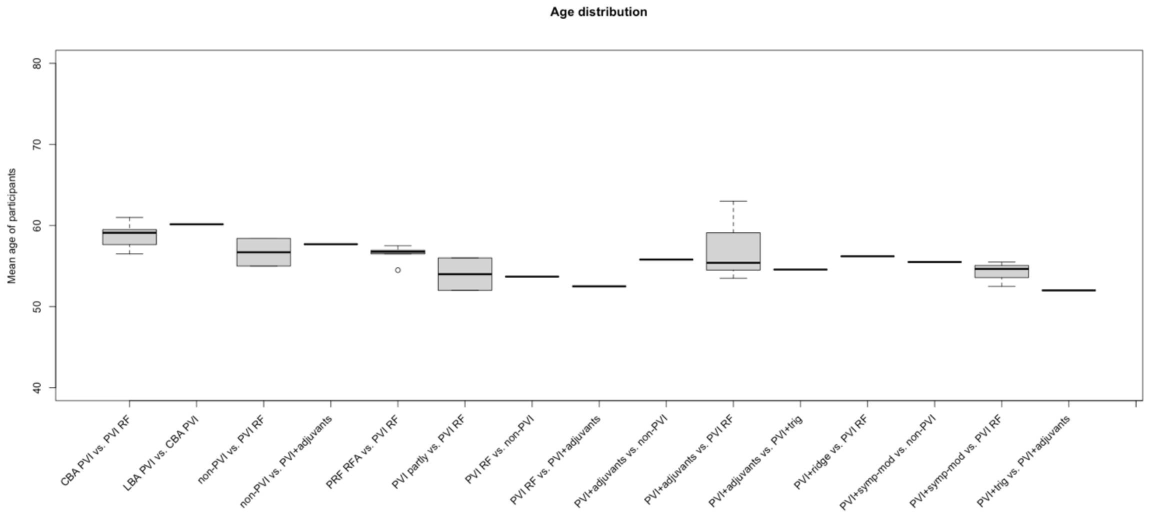

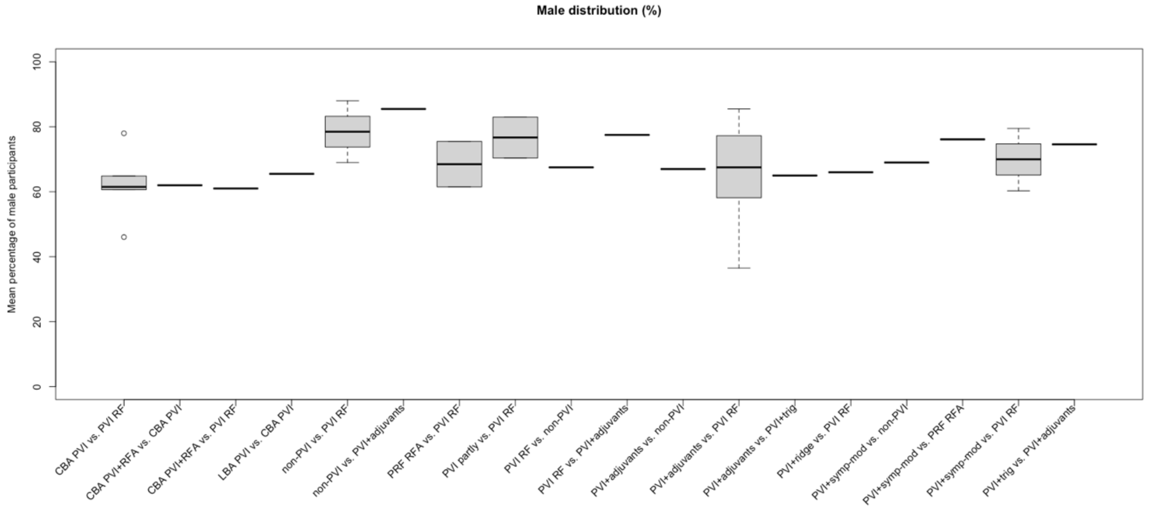

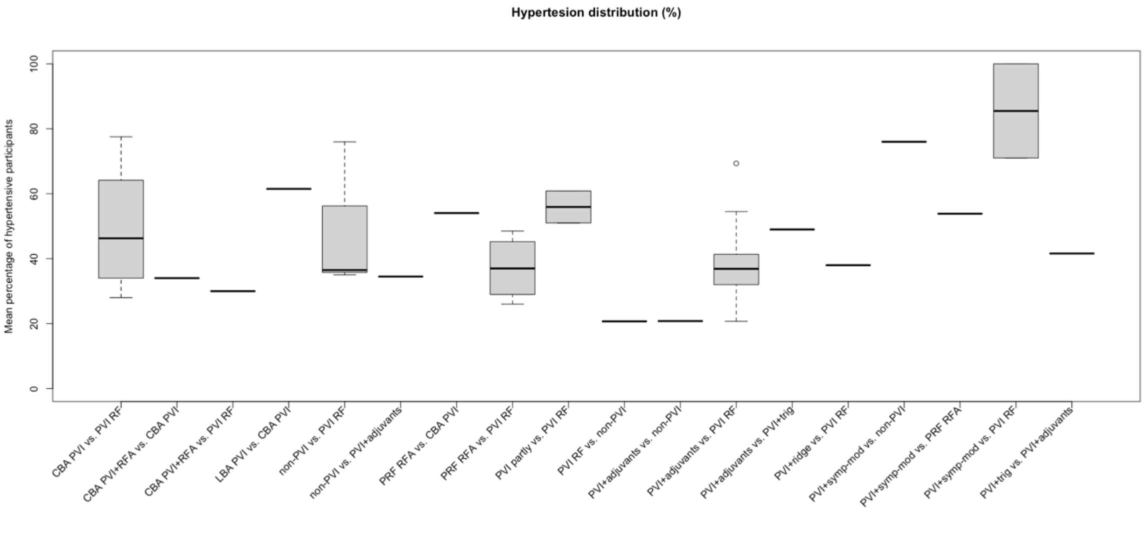
Hypertesion distribution (%)
Mean percentage of hypertensive participants
0
20
40
60
80
100
CBA PVI vs. PVI RF
CBA PVI+RFA vs. CBA PVI
CBA PVI+RFA vs. PVI RF
LBA PVI vs. CBA PVI
non-PVI vs. PVI RF
non-PVI vs. PVI+adjuvants
PRF RFA vs. CBA PVI
PRF RFA vs. PVI RF
PVI partly vs. PVI RF
PVI RF vs. non-PVI
PVI+adjuvants vs. non-PVI
PVI+adjuvants vs. PVI RF
PVI+adjuvants vs. PVI+trig
PVI+ridge vs. PVI RF
PVI+symp-mod vs. non-PVI
PVI+symp-mod vs. PRF RFA
PVI+symp-mod vs. PVI RF
PVI+trig vs. PVI+adjuvants

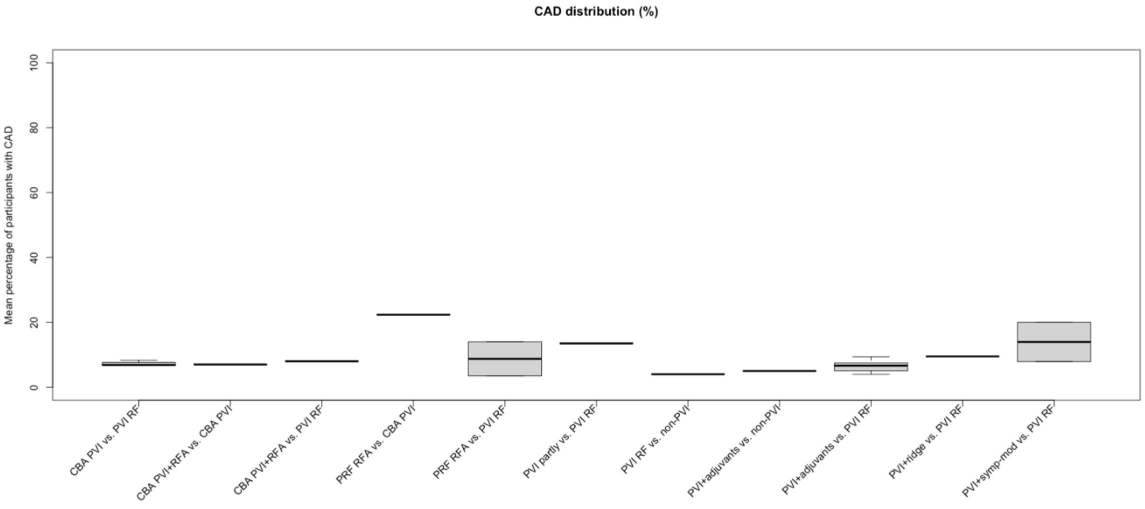
CAD distribution (%)
Mean percentage of participants with CAD
0
20
40
60
80
100
CBA PVI vs. PVI RF
CBA PVI+RFA vs. CBA PVI
CBA PVI+RFA vs. PVI RF
PRF RFA vs. CBA PVI
PRF RFA vs. PVI RF
PVI partly vs. PVI RF
PVI RF vs. non-PVI
PVI+adjuvants vs. non-PVI
PVI+adjuvants vs. PVI RF
PVI+ridge vs. PVI RF
PVI+symp-mod vs. PVI RF

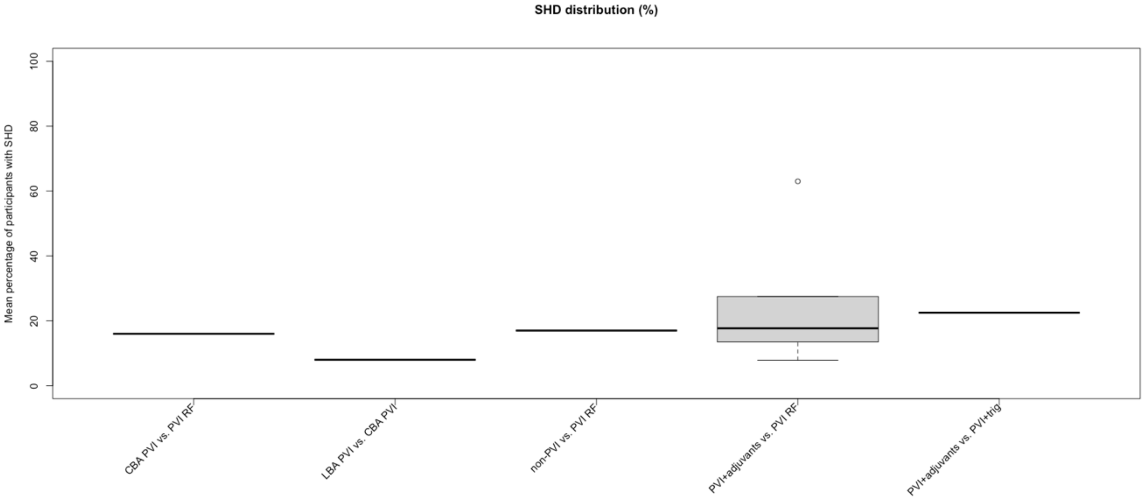
SHD distribution (%)
Mean percentage of participants with SHD
0
20
40
60
80
100
CBA PVI vs. PVI RF
LBA PVI vs. CBA PVI
non-PVI vs. PVI RF
PVI+adjuvants vs. PVI RF
PVI+adjuvants vs. PVI+trig

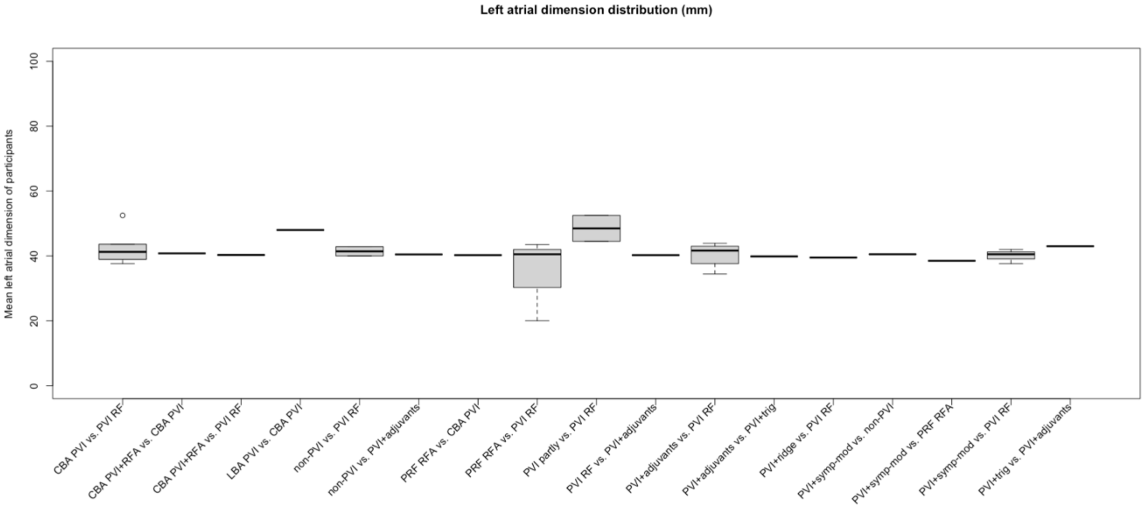

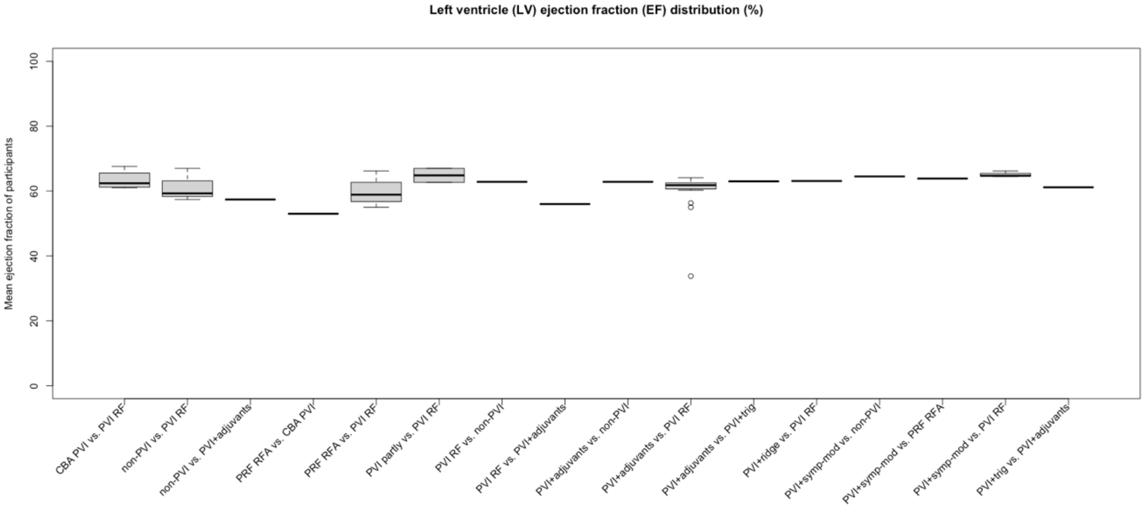

Additional transitivity boxplots for all comparisons including also comparisons with AADs:

As above, we show boxplots for the distributions of mean age, percentage of males, presence of hypertension, coronary artery disease (CAD), structural heart disease (SHD), left atrial dimensions and left ventricle (LV) ejection fraction (EF) across the available direct comparisons including also comparisons with AADs. We can note that some of the comparisons involving AADs are responsible for wider distributions and more deviating behaviour.

Age distribution

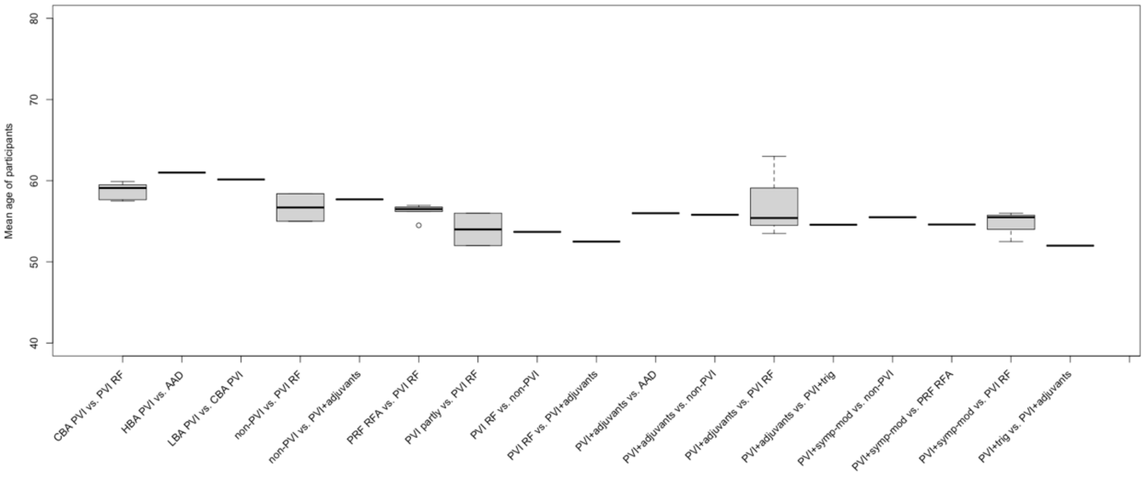

Male distribution (%)

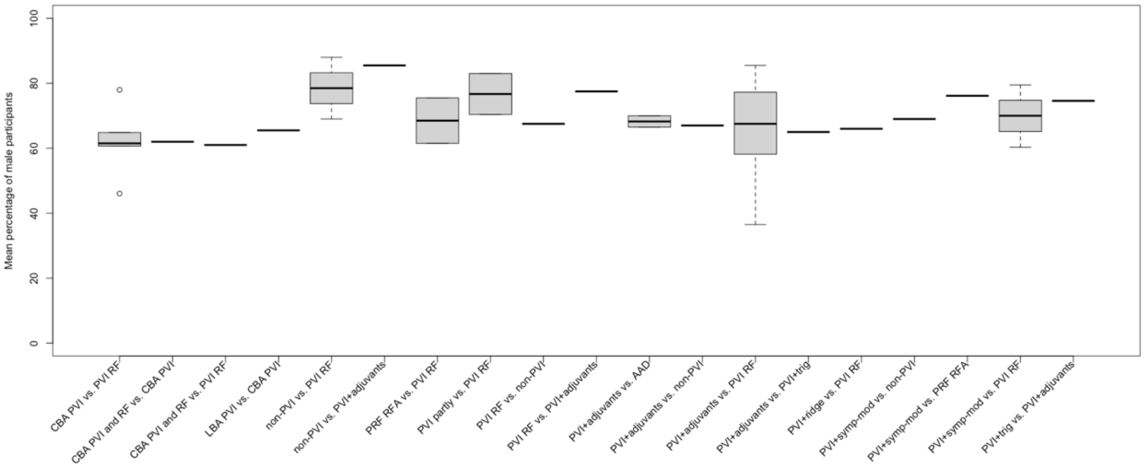

Hypertesion distribution (%)

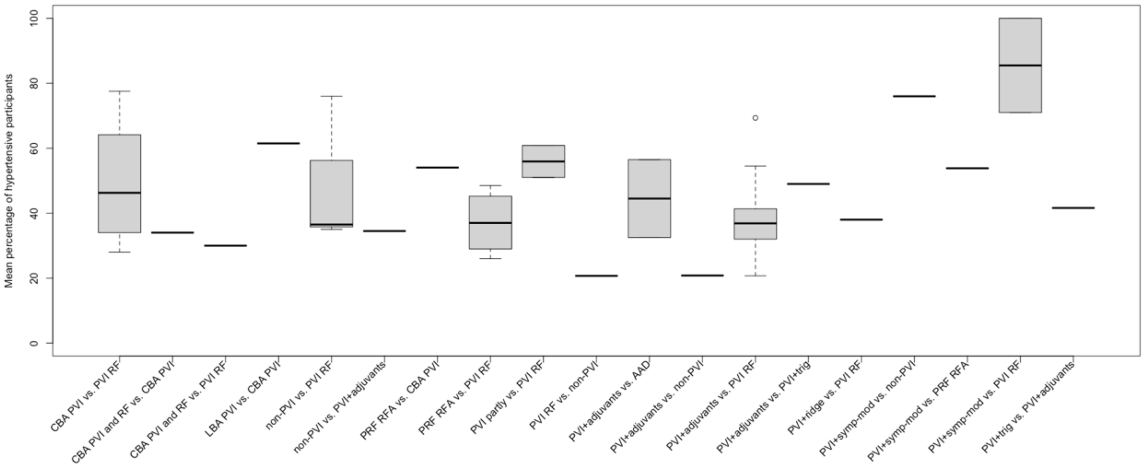

SHD distribution (%)

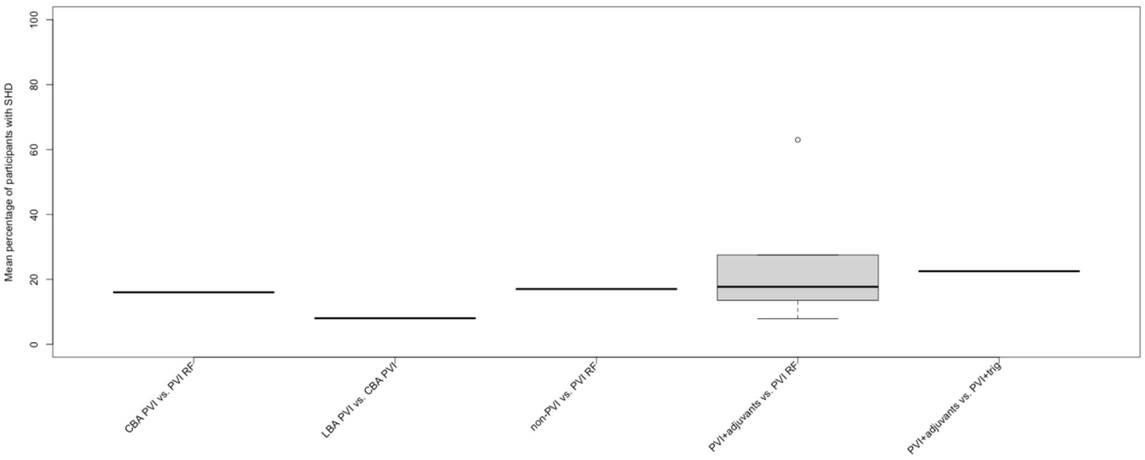

CAD distribution (%)

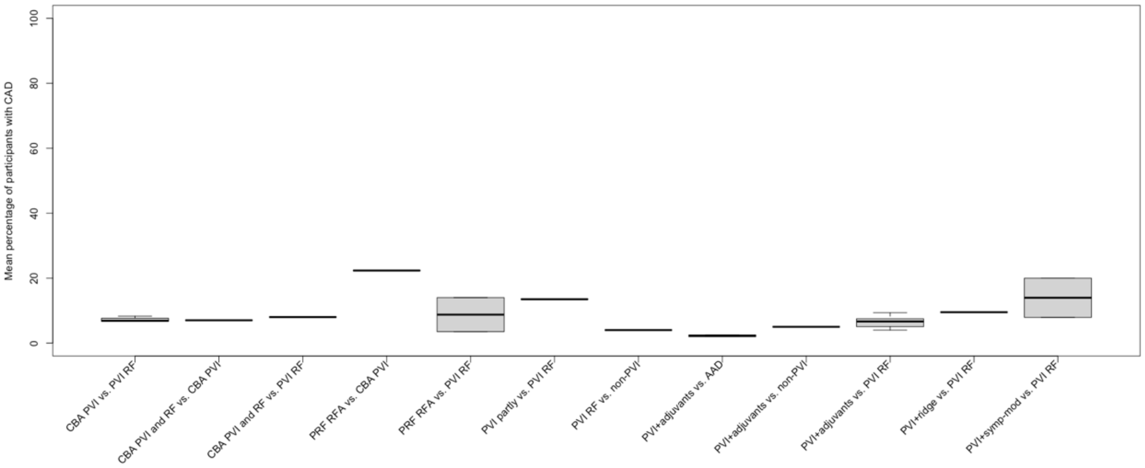

Left atrial dimension distribution (mm)

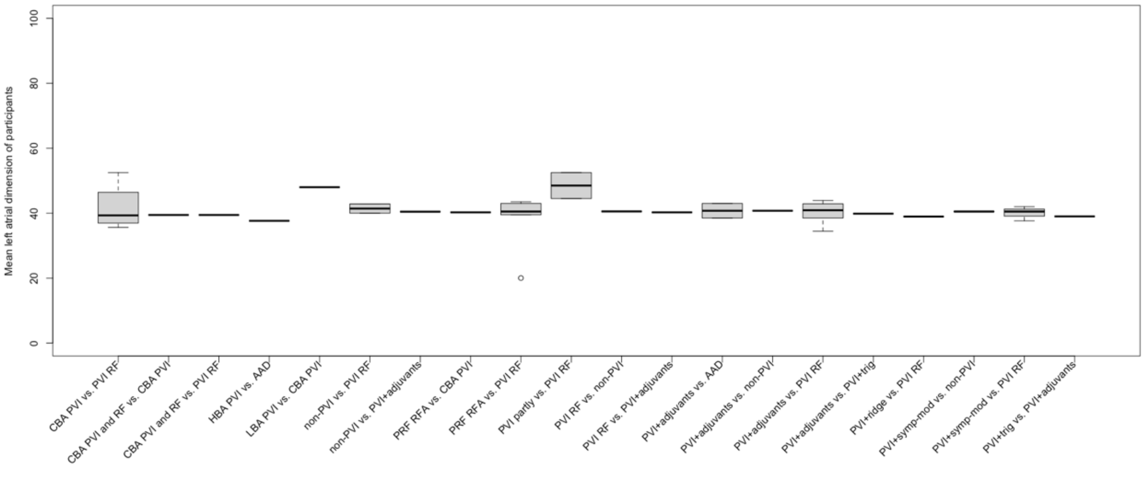

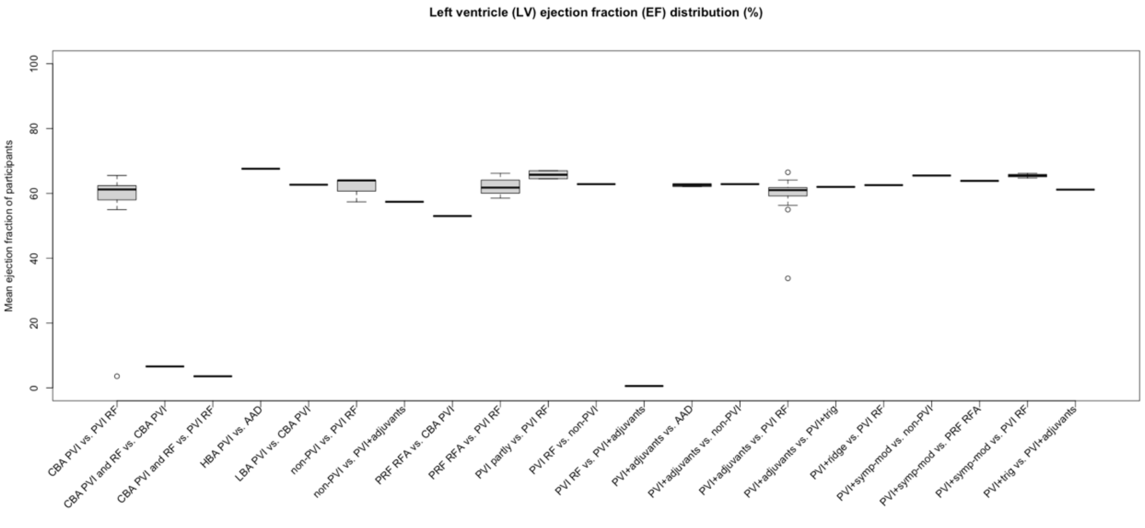
Left ventricle (LV) ejection fraction (EF) distribution (%)
Mean ejection fraction of participants
0
20
40
60
80
100
CBA PVI vs. PVI RF
CBA PVI and RF vs. CBA PVI
CBA PVI and RF vs. PVI RF
HBA PVI vs. AAD
LBA PVI vs. CBA PVI
non-PVI vs. PVI RF
non-PVI vs. PVI+adjuvants
PRF RFA vs. CBA PVI
PRF RFA vs. PVI RF
PVI partly vs. PVI RF
PVI RF vs. non-PVI
PVI RF vs. PVI+adjuvants
PVI+adjuvants vs. AAD
PVI+adjuvants vs. non-PVI
PVI+adjuvants vs. PVI RF
PVI+adjuvants vs. PVI+trig
PVI+ridge vs. PVI RF
PVI+symp-mod vs. non-PVI
PVI+symp-mod vs. PRF RFA
PVI+symp-mod vs. PVI RF
PVI+trig vs. PVI+adjuvants

Section S7. ADDITIONAL RESULTS FROM PAIRWISE AND NETWORK META-ANALYSIS

A random effects NMA model with common heterogeneity was employed. We fitted models for pairwise and network meta-analysis using the R library 'netmeta' (R version 4.0.2).
Additional results contained in this Appendix: league tables of RRs from NMA for each outcome (eTable2, eTable3, eTable4), additional NMA forest plots for efficacy and safety with AAD as reference treatment (eFigure2) and P-scores for ranking treatments for efficacy and safety (eFigure3).

Table S3. Relative risk ratios for efficacy estimated from the network meta-analysis (lower triangle) and pairwise meta-analysis (upper triangle) comparing every pair of the 13 interventions.

PVI RF	1.00 (0.81; 1.25)	.	0.98 (0.71; 1.35)	0.85 (0.53; 1.36)	2.21 (1.16; 4.22)	1.60 (1.11; 2.29)	1.23 (1.00; 1.50)	.	0.80 (0.44; 1.44)	0.62 (0.44; 0.89)
1.00 (0.81; 1.23)	CBA PVI	0.89 (0.37; 2.10)	1.00 (0.52; 1.94)	.	1.39 (0.70; 2.75)	.	.	.	.	.
0.88 (0.36; 2.15)	0.89 (0.37; 2.10)	LBA PVI	.	.	.	.	.	.	.	.
0.95 (0.72; 1.26)	0.96 (0.69; 1.32)	1.08 (0.43; 2.72)	PRF RFA	.	.	2.06 (0.93; 4.55)	.	.	.	.
0.85 (0.53; 1.36)	0.85 (0.51; 1.43)	0.96 (0.35; 2.63)	0.89 (0.52; 1.54)	PVI partly	.	.	.	.	.	.
1.81 (1.00; 3.30)	1.82 (0.99; 3.32)	2.05 (0.71; 5.89)	1.90 (0.99; 3.66)	2.13 (0.99; 4.56)	CBA PVI + RFA	.	.	.	.	.
1.57 (1.14; 2.18)	1.57 (1.07; 2.31)	1.78 (0.69; 4.58)	1.65 (1.11; 2.45)	1.85 (1.04; 3.27)	0.87 (0.44; 1.71)	PVI+ symp-mod	.	.	.	0.49 (0.26; 0.92)
1.26 (1.03; 1.54)	1.26 (0.95; 1.69)	1.43 (0.57; 3.55)	1.32 (0.94; 1.86)	1.48 (0.89; 2.47)	0.70 (0.37; 1.31)	0.80 (0.55; 1.17)	PVI+adjuvants	0.97 (0.62; 1.54)	.	0.35 (0.19; 0.63)
1.23 (0.75; 2.02)	1.23 (0.72; 2.11)	1.39 (0.50; 3.85)	1.29 (0.73; 2.28)	1.44 (0.73; 2.86)	0.68 (0.31; 1.48)	0.78 (0.43; 1.41)	0.97 (0.62; 1.54)	PVI+trig	.	.
0.80 (0.44; 1.44)	0.80 (0.43; 1.49)	0.90 (0.31; 2.61)	0.83 (0.43; 1.60)	0.93 (0.44; 1.99)	0.44 (0.19; 1.02)	0.51 (0.26; 0.99)	0.63 (0.34; 1.17)	0.65 (0.30; 1.40)	PVI+ridge	.
0.61 (0.44; 0.83)	0.61 (0.42; 0.89)	0.69 (0.27; 1.76)	0.64 (0.42; 0.96)	0.71 (0.40; 1.26)	0.33 (0.17; 0.66)	0.39 (0.25; 0.58)	0.48 (0.34; 0.68)	0.49 (0.28; 0.88)	0.76 (0.39; 1.49)	non-PVI

NMA results (RR<1 favour the treatment in the column)
Heterogeneity: τ^2=0.0567

Table S4. Relative risk ratios estimated for safety from the network meta-analysis (lower triangle) and pairwise meta-analysis (upper triangle) comparing every pair of the 12 interventions.

PVI RF	0.98 (0.71; 1.34)	.	1.45 (0.49; 4.32)	0.21 (0.01; 4.24)	1.37 (0.32; 5.91)	1.19 (0.44; 3.20)	0.88 (0.48; 1.62)	.	2.50 (0.49; 12.63)	2.48 (0.69; 8.88)
1.00 (0.74; 1.37)	CBA PVI	0.70 (0.26; 1.85)	0.71 (0.17; 3.07)	.	1.35 (0.31; 5.84)	.	.	.	.	.
0.70 (0.25; 1.95)	0.70 (0.26; 1.85)	LBA PVI	.	.	.	.	.	.	.	.
1.21 (0.52; 2.84)	1.21 (0.51; 2.88)	1.73 (0.47; 6.40)	PRF RFA	.	.	0.34 (0.01; 8.13)	.	.	.	.
0.21 (0.01; 4.24)	0.21 (0.01; 4.29)	0.29 (0.01; 7.17)	0.17 (0.01; 3.92)	PVI partly	.	.	.	.	.	.
1.36 (0.37; 5.04)	1.36 (0.37; 5.02)	1.95 (0.38; 9.95)	1.12 (0.24; 5.28)	6.61 (0.25; 178.37)	CBA PVI + RFA	.	.	.	.	.
1.08 (0.42; 2.79)	1.08 (0.40; 2.92)	1.55 (0.38; 6.23)	0.89 (0.26; 3.03)	5.26 (0.22; 125.15)	0.80 (0.16; 4.00)	PVI+ symp-mod	.	.	.	.
0.88 (0.48; 1.62)	0.88 (0.45; 1.74)	1.26 (0.38; 4.15)	0.73 (0.26; 2.07)	4.29 (0.20; 93.85)	0.65 (0.15; 2.75)	0.82 (0.26; 2.51)	PVI+ adjuvants	1.32 (0.74; 2.33)	.	.
1.16 (0.50; 2.68)	1.16 (0.48; 2.83)	1.66 (0.44; 6.23)	0.96 (0.29; 3.16)	5.65 (0.25; 130.27)	0.86 (0.18; 4.04)	1.07 (0.30; 3.80)	1.32 (0.74; 2.33)	PVI+trig	.	.
2.50 (0.49; 12.63)	2.49 (0.48; 12.97)	3.57 (0.53; 24.28)	2.06 (0.33; 12.83)	12.14 (0.39; 375.21)	1.84 (0.23; 14.73)	2.31 (0.35; 15.07)	2.83 (0.50; 15.96)	2.15 (0.35; 13.29)	PVI+ridge	.
2.48 (0.69; 8.88)	2.48 (0.67; 9.18)	3.55 (0.69; 18.19)	2.05 (0.44; 9.46)	12.06 (0.45; 321.04)	1.82 (0.29; 11.33)	2.29 (0.47; 11.21)	2.81 (0.68; 11.53)	2.13 (0.47; 9.79)	0.99 (0.13; 7.80)	non-PVI

NMA results (RR<1 favour the treatment in the column)

Heterogeneity: τ^2=0.00

Table S5. Relative risk ratios for procedural time estimated from the network meta-analysis (lower triangle) and pairwise meta-analysis (upper triangle) comparing every pair of the 10 interventions.

PVI RF	-0.05 (-0.53; 0.43)	.	1.05 0.69; 1.41)	0.84 (0.27; 1.41)	.	-0.04 (-0.81; 0.74)	.	-0.41 (-0.65; -0.16)	-0.29 (-0.88; 0.31)
0.14 (-0.29; 0.57)	CBA PVI	-0.13 (-0.92; 0.65)	0.08 (-0.84; 1.01)	.	.	.	.	.	.
0.01 (-0.89; 0.90)	-0.13 (-0.92; 0.65)	LBA PVI	.	.	.	.	.	.	.
0.94 (0.60; 1.28)	0.80 (0.30; 1.30)	0.93 (0.00; 1.86)	PRF RFA	.	-0.35 (-1.13; 0.44)	.	.	.	.
0.84 (0.27; 1.41)	0.70 (-0.02; 1.42)	0.83 (-0.23; 1.89)	-0.10 (-0.77; 0.56)	PVI partly	.	.	.	.	.
0.59 (-0.26; 1.45)	0.45 (-0.48; 1.39)	0.59 (-0.63; 1.80)	-0.35 (-1.13; 0.44)	-0.24 (-1.28; 0.79)	PVI+ symp-mod	.	.	.	.
-0.03 (-0.81; 0.74)	-0.18 (-1.07; 0.71)	-0.04 (-1.23; 1.14)	-0.98 (-1.83; -0.13)	-0.87 (-1.84; 0.09)	-0.63 (-1.79; 0.53)	PVI+ridge	.	.	.
-0.23 (-0.85; 0.38)	-0.38 (-1.12; 0.37)	-0.24 (-1.33; 0.84)	-1.18 (-1.87; -0.48)	-1.07 (-1.91; -0.23)	-0.83 (-1.88; 0.22)	-0.20 (-1.19; 0.79)	PVI+trig	-0.18 (-0.74; 0.38)	.
-0.41 (-0.65; -0.17)	-0.55 (-1.05; -0.06)	-0.42 (-1.35; 0.51)	-1.35 (-1.77; -0.93)	-1.25 (-1.87; -0.63)	-1.01 (-1.90; -0.11)	-0.38 (-1.19; 0.44)	-0.18 (-0.74; 0.38)	PVI+adjuvants	0.64 (-0.19; 1.46)
-0.13 (-0.68; 0.43)	-0.27 (-0.97; 0.43)	-0.14 (-1.19; 0.92)	-1.07 (-1.72; -0.42)	-0.97 (-1.77; -0.17)	-0.72 (-1.74; 0.30)	-0.09 (-1.05; 0.86)	0.11 (-0.70; 0.91)	0.28 (-0.29; 0.86)	non-PVI

MD (hours scale) <0 favours the treatment in the column

Heterogeneity: τ^2= 0.151

Figure S1: P-scores for the two primary outcomes

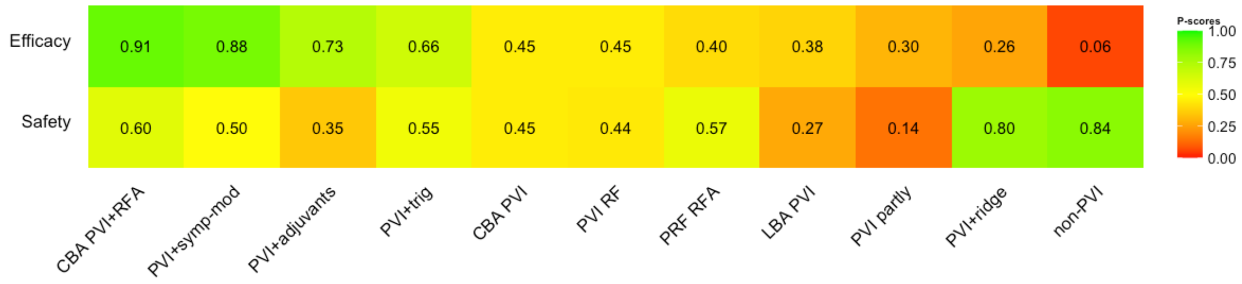

The interpretation of the ranking of strategies concerning the safety outcome (outcome 2), according to the P-scores, must be treated with cautious due to the high uncertainty inspected.

Section S8. EVALUATION OF INCONSISTENCY

Consistency refers to the statistical agreement between direct and indirect evidence in the network. The presence of inconsistency in the network has been evaluated with the global design-by-treatment interaction model and with the net-split approach.
The design-by-treatment interaction model is a general model for inconsistency that provides a single inference test, based on the χ^2 test, about the plausibility of assuming consistency for the entire network. However, inconsistency models may have low power and may sometimes fail to detect inconsistency.

Table S6. Design-by-treatment interaction test, with global p-value, Q statistic and degrees of freedom for each outcome.

Outcome	p-value	Q (df)
**Efficacy**	0.1020	11.80 (7)
**Safety**	0.7978	1.03 (3)
**Procedural time**	0.1387	5.50 (3)

The net-split approach splits the network estimates into the contribution of direct and indirect evidence, which allows us to check for inconsistency in specific comparisons in our network.

Node-splitting method (p-values<0.05 indicate disagreement between direct and indirect estimates)

Efficacy**:**

comparisons with p-values<0.05: 0% of total comparisons
comparisons with p-values<0.10: 0% of total comparisons

Safety**:**

comparisons with p-values<0.05: 0% of total comparisons
comparisons with p-values<0.10: 0% of total comparisons

Procedural time**:**

comparisons with p-values<0.05: 0% of total comparisons
comparisons with p-values<0.10: 6% of total comparisons

Table S7. Results of the inconsistency net-split approach for all outcomes. For each comparison the direct and indirect estimates are provided along with the respective z-values and p-values of differences. P-values<0.10 indicate significant disagreement between direct and indirect evidence (in red), while 0.10<p-value<0.15 may refer to moderate concerns (in yellow).

	Comparisons	NMA	direct	indirect	z	p-value
**Efficacy**						
	CBA PVI vs CBA PVI+RF	1.8154	1.3860	4.6405	-1.6330	0.1025
	CBA PVI vs PRF RFA	0.9553	1.0000	0.9415	0.1557	0.8763
	CBA PVI vs PVI RFA	1.0003	0.9953	1.0572	-0.1557 -	0.8763
	CBA PVI+RFA vs PVI RF	0.5510	0.4517	1.9258	1.6330	0.1025
	non-PVI vs PVI RF	1.6468	1.6015	1.8374	-0.3417	0.7326
	non-PVI vs PVI+adjuvants	2.0814	2.8799	1.7448	1.3401	0.1802
	non-PVI vs PVI+symp-mod	2.5910	2.0476	3.1077	-0.9754	0.3294
	PRF RFA vs PVI RF	1.0472	1.0203	1.1277	-0.3108	0.7559
	PRF RFA vs PVI+symp-mod	1.6476	2.0606	1.5288	0.6387	0.5230
	PVI+adjuvants vs PVI RF	0.7912	0.8146	0.3131	1.6080	0.1078
	PVI+symp-mod vs PVI RF	0.6356	0.6269	0.6733	-0.1695	0.8654
**Safety**						
	CBA PVI vs CBA PVI+RFA	1.3573	1.3504	1.3843	-0.0149	0.9881
	CBA PVI vs PRF RFA	1.2098	0.7143	1.6169	-0.8828	0.3774
	CBA PVI vs PVI RF	0.9971	1.0218	0.4514	0.8828	0.3774
	CBA PVI+RF vs PVI RF	0.7347	0.7310	0.7494	-0.0149	0.9881
	PRF RFA vs PVI RF	0.8242	0.6876	1.0932	-0.5218	0.6018
	PRF RFA vs PVI+symp-mod	0.8928	0.3433	1.0551	-0.6415	0.5212
	PVI+symp-mod vs PVI RF	0.9232	0.8409	2.5846	-0.6415	0.5212
**Procedural time**						
	CBA PVI vs PRF RFA	0.7993	0.0833	1.1011	-1.8114	**0.0701**
	CBA PVI vs RF PVI	-0.1421	0.0517	-0.9661	-1.8114	**0.0701**
	non-PVI vs PVI+adjuvants	-0.2832	-0.6367	0.0584	-1.1801	0.2379
	non-PVI vs PVI RF	0.1272	0.2885	-0.9819	1.4921	0.1357
	PRF RFA vs PVI RF	-0.9415	-1.0494	-0.0316	-1.8114	**0.0701**
	PVI+adjuvants vs PVI RF	0.4103	0.7147	-0.3079	-0.2656	0.7906

Figure S2. Forest plots of the net-split approach separating direct and indirect evidence for efficacy.

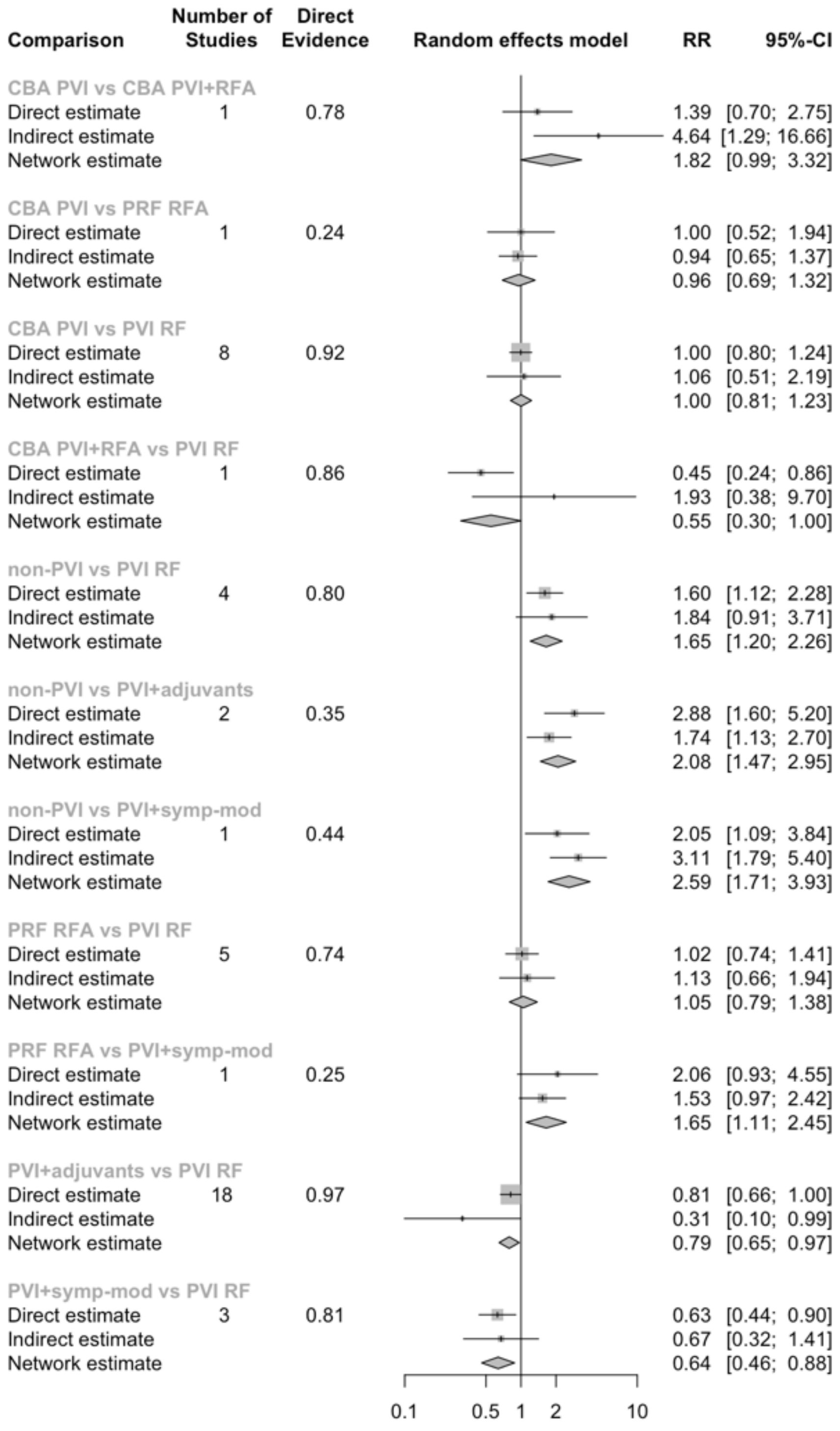

Figure S3. Forest plots of the net-split approach separating direct and indirect evidence for safety.

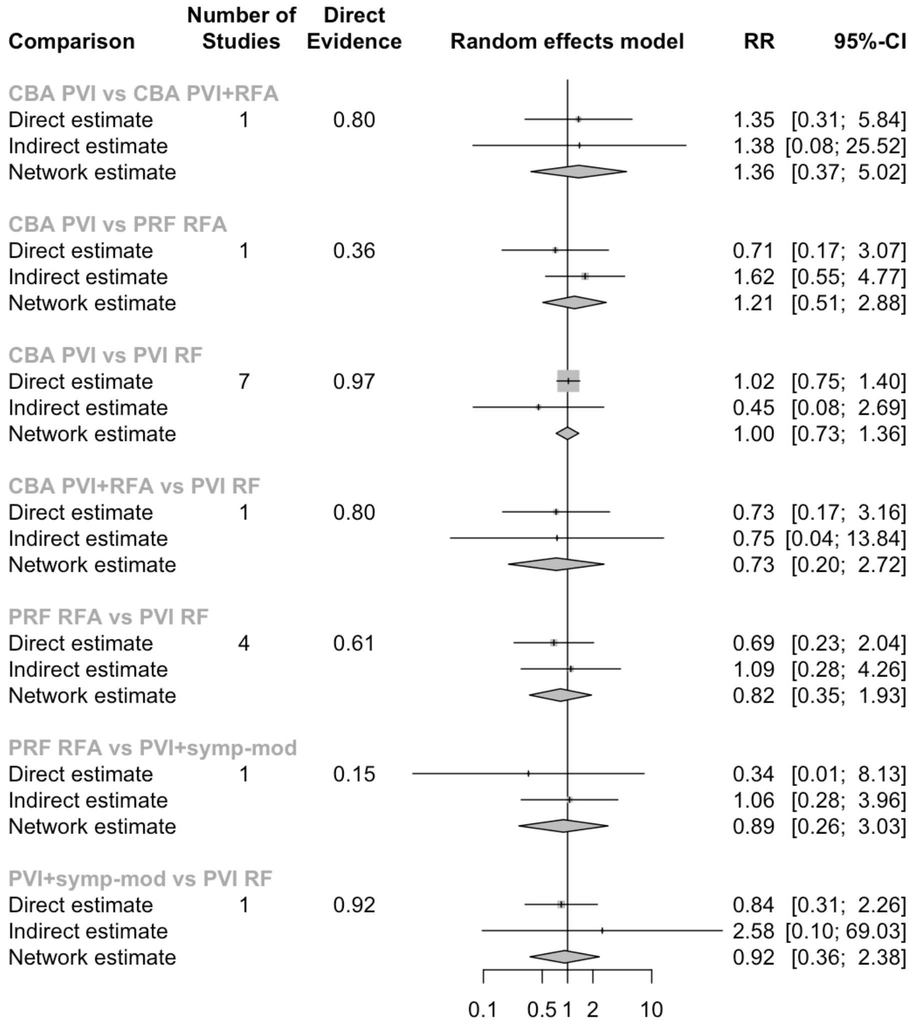

Figure S4. Forest plots of the net-split approach separating direct and indirect evidence for procedural time.

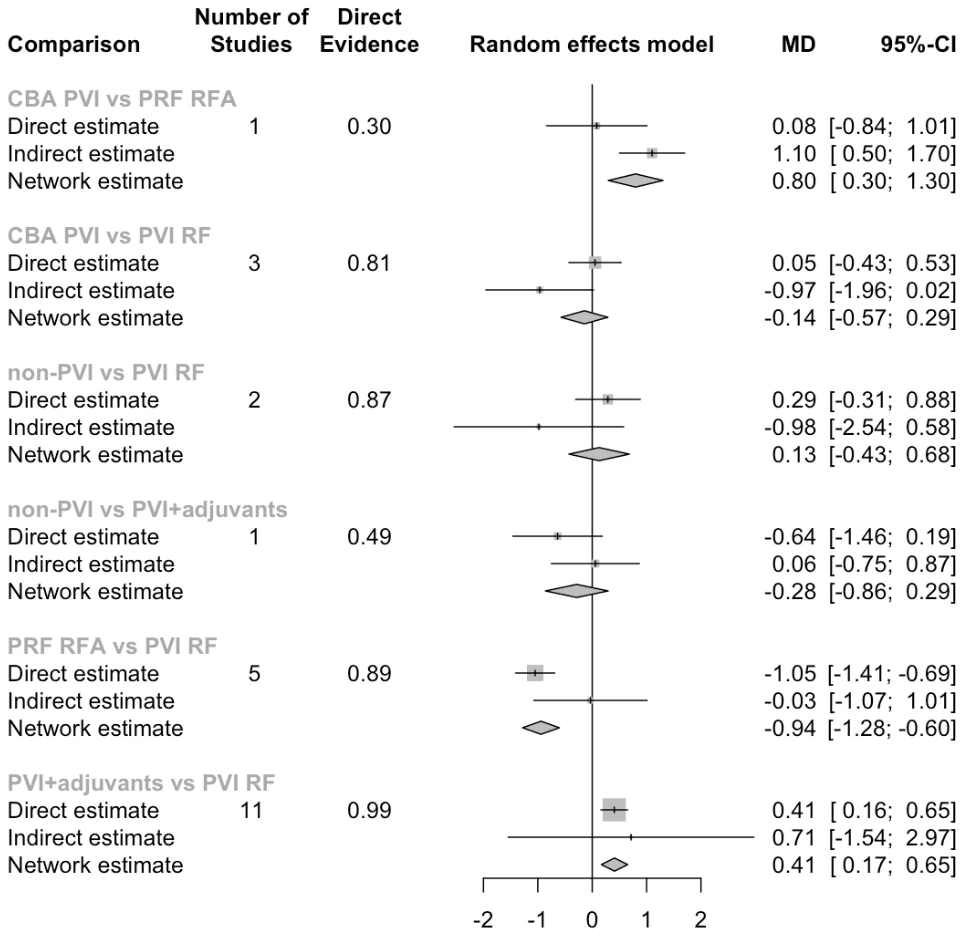

Section S9. INVESTIGATION OF SMALL-STUDY EFFECTS

Comparison-adjusted funnel plots were used to assess the presence of small-study effects, i.e. whether important differences in treatment effect estimates between more precise and less precise studies exist.

eFigure7, eFigure8 and eFigure9 depict the comparison-adjusted funnel plots for each outcome. The horizontal axis represents the difference between the study effect and the summary effect for each comparison, while on the vertical axis is the standard error of the observed effect size, in reversed order (precise studies at the top). Asymmetry in the plot indicates concern for small study effects.

We used comparison-adjusted funnel plots for all active strategies against PVI RF (control treatment). For efficacy, the plot appears quite symmetric around the line of no effect, suggesting the absence of small-study effects. For safety, there is a slight asymmetry while for procedural time the plot reveals the presence of important heterogeneity.

Figure S5. Comparison-adjusted funnel plot for efficacy

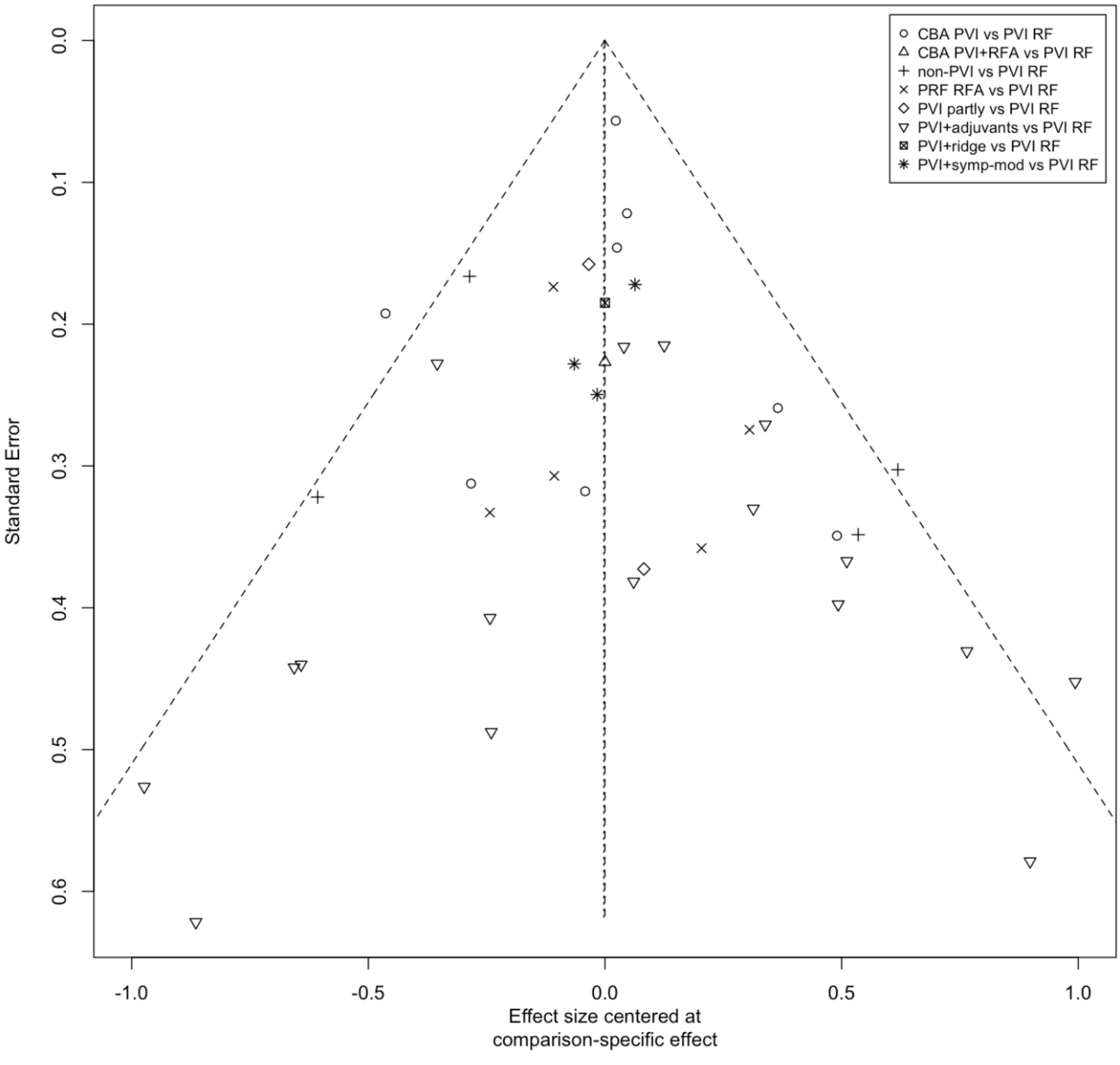

Figure S6. Comparison-adjusted funnel plot for safety.

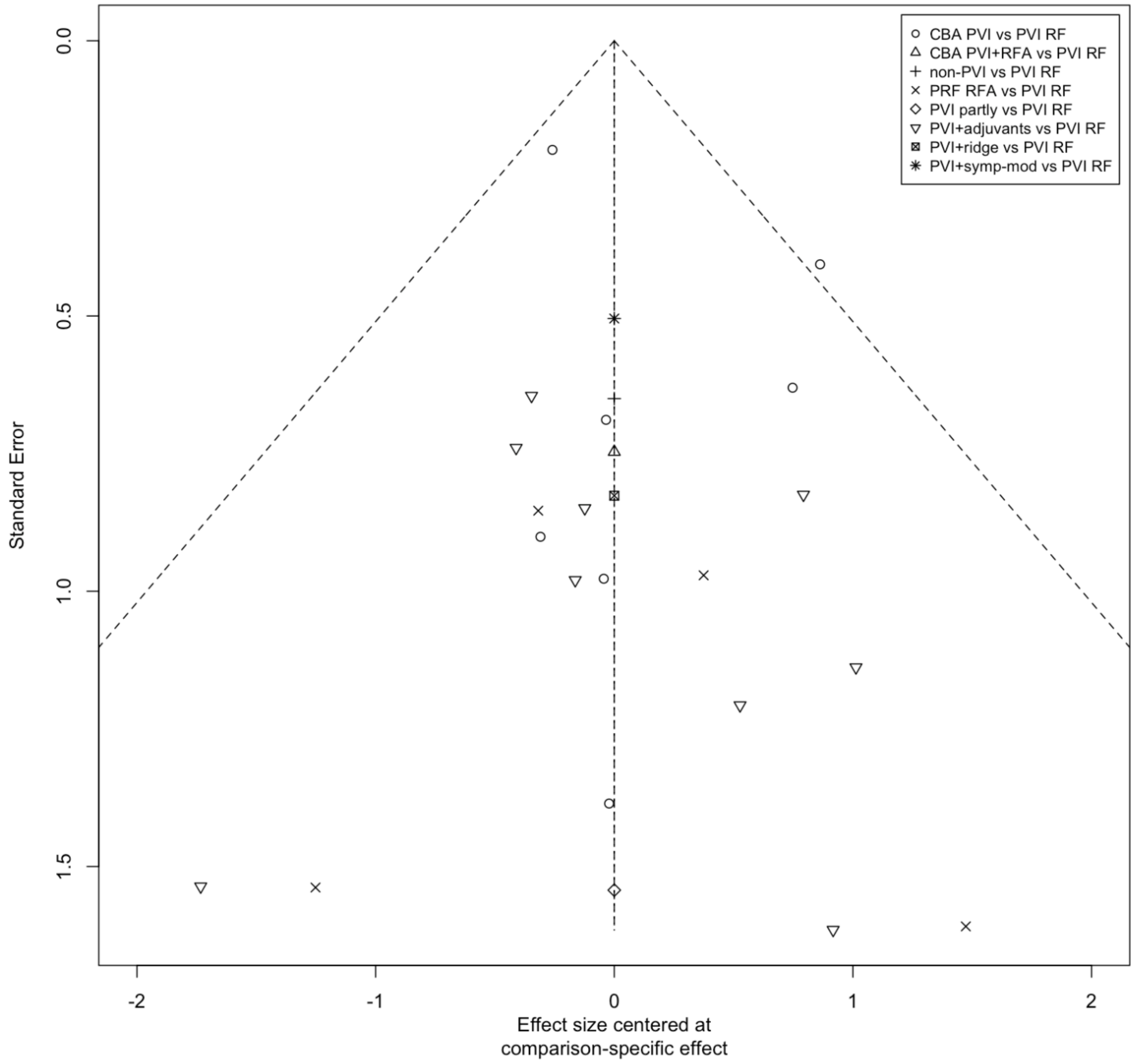

Figure S7. Comparison-adjusted funnel plot for procedural time.

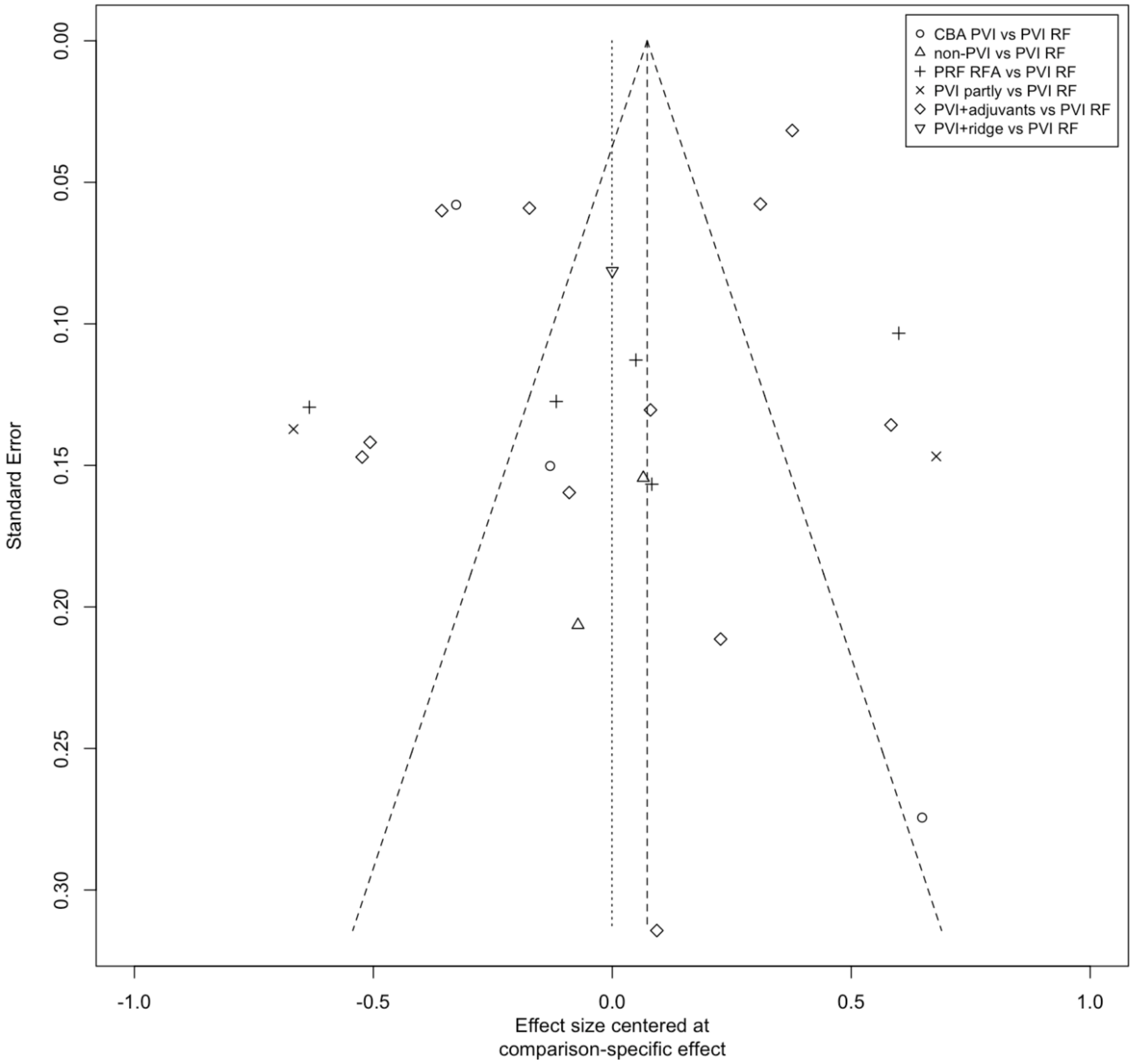

Section S10. SUBGROUP ANALYSES

1 Depending on AF detection device (ECGs with recording times≥7 days vs others)

ECGs with recording times ≥7 (14 RCTs)

τ^2=0.0197

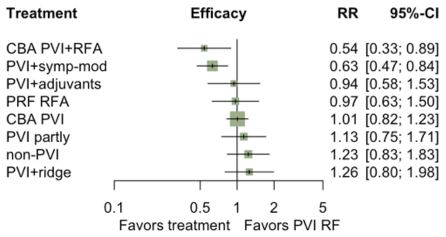

ECGs with recording times <7 (29 RCTs)

τ^2=0.136

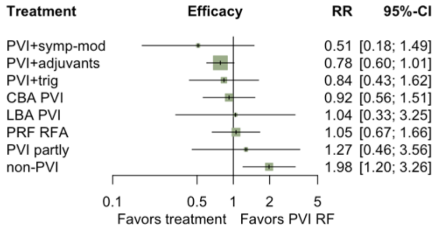

τ^2=0

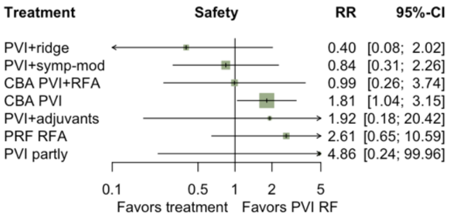

τ^2=0

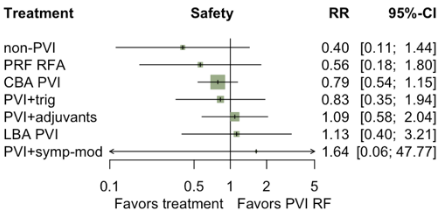

2 Depending on AAD or reablation allowance during the follow-up

Not allowing for ADD (33 RCTs)

Allowing for ADD (10 RCTs)

τ^2=0.043

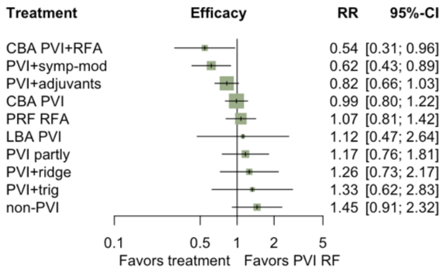

τ^2=0.256

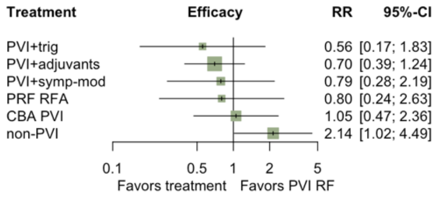

τ^2=0

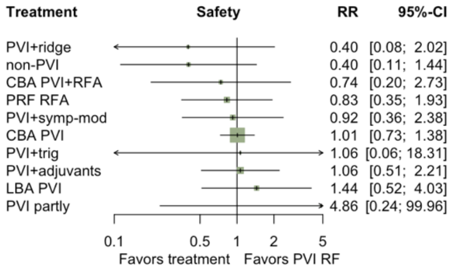

τ^2=0

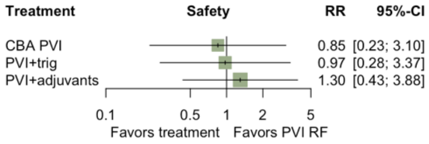

3 Depending on follow-up duration (≥12 months vs < 12 months)

Follow-up ≥ 12 months (39 studies)

Follow-up < 12 months (4 studies)

τ^2=0.0573

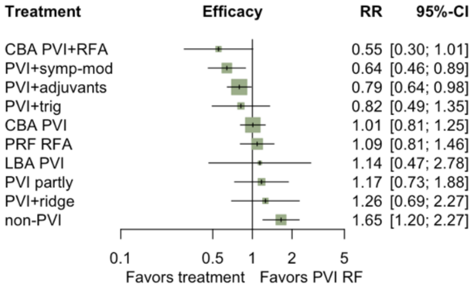

τ^2=0.857

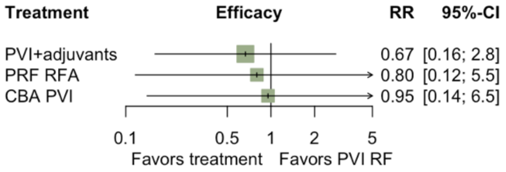

τ^2=0.00

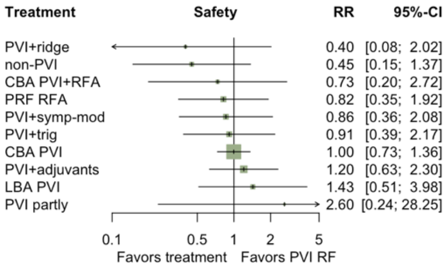

τ^2= NA

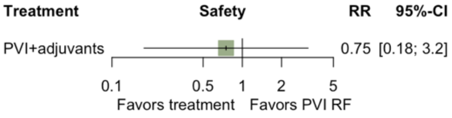

(2 studies had missing safety outcome)

4 Depending on publication year ($\geq$2011 vs. before)

Publication year $\geq$ 2011 (32 studies)

τ^2=0.043

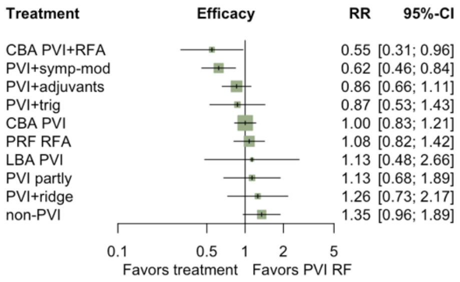

τ^2=0

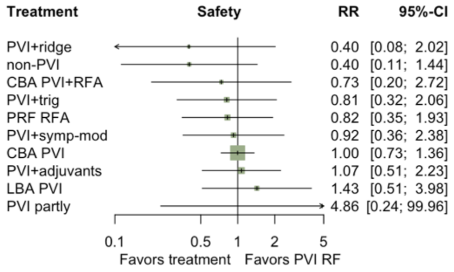

Publication year < 2011 (11 studies)

τ^2=0.0827

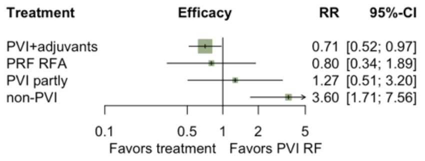

τ^2=0

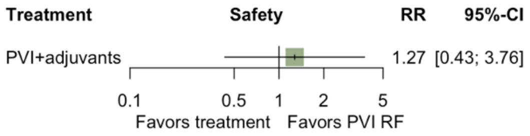

(10 studies had missing safety outcome)

Section S11. META-REGRESSION

We fitted network random-effects meta-regression models, with common consistent coefficients, to investigate the impact of some prespecified variables that may act as effect modifiers on the primary outcome efficacy.

For each study $i = 1, \dots, N$ let r_{ik} be the number of events in arm $k = 1, \dots, K$ and n_{ik} the sample size in arm k. As we have binary outcome data, we have:

$$r_{ik} \sim \mathrm{Binom}(\pi_{i,k}, n_{ik}) \quad \text{(Binomial likelihood)},$$

with the probability $\pi_{i,k}$ given by

$$\mathrm{logit}(\pi_{i,k}) = \mu_i \quad if\ k = 1$$

$$\mathrm{logit}(\pi_{i,k}) = \mu_i + \theta_{i,k} \quad if\ k \geq 2$$

$$\theta_{i,k} = \delta_{i,1k} + \beta_{t_{i1},t_{ik}} x_i$$

where $\beta_{t_{i1},t_{ik}} = \beta_{1,t_{ik}} - \beta_{1,t_{i1}}$ is the difference in the relative treatment effect of $t_{i,k}\ vs\ t_{i,1}$ per increase of one unit in the covariate x_i

In a random-effects model, $\delta_{i,1k}$, for each $k \geq 2$, represents the trial-specific relative treatment effect when the covariate is 0 and is assumed to follow a normal distribution

$$\delta_{i,1k} \sim N(d_{t_{i1},t_{i,k}}, \tau^2)$$

with $d_{t_{i1},t_{i,k}} = d_{1,t_{i,k}} - d_{1,t_{i,1}}$ being the mean relative treatment effect of $t_{i,k}\ \ vs\ \ t_{i,1}$ when the covariate is 0.

As we use a common regression coefficient model, we assume

$$\beta_{t_{i1},t_{ik}} = \beta$$

For each covariate analysed, the model is estimated within a Bayesian framework (R library gemtc version 0.8.7) using the following priors:

$$\mu_i \sim N(0{,}1000), d_{1,t_{i,k}} \sim N(0{,}1000), \beta \sim N(0{,}1000)$$

$$\tau \sim U(0{,}5)$$

We included as covariates in separate network meta-regression models the following variables: age, percentage of males, publication year, presence of hypertension, coronary artery disease (CAD), structural heart disease (SHD), left atrial dimensions, duration of follow-up and usage of AF detection device. The impact of each covariate was assessed in independent univariate analyses. Continuous covariates were cantered at the mean (subtracting the mean covariate value from each covariate). Not all the studies reported all the covariates of interest, so if a study was missing a covariate, it was omitted from that regression model. Only effect modifiers which non-missing outcomes for at least 10 studies were retained. Results are reported in eTable 6.

For each meta-regression analysis, regression coefficients are reported alongside their credible intervals (CrI): all CrIs contain zero (not significative coefficient) except for Age. This suggests that most of the effect modifiers analysed seem to impact on our NMA results while Age seems to have an impact and it also is the only variable associated with a (large) reduction in heterogeneity.

Table S8. Meta-regression coefficients, alongside Credible Intervals and percentage reduction in heterogeneity for efficacy outcome.

Covariate	N studies	Coefficient	95% CrI	$\%\tau^2$ reduction
Age	33	0.56215	(0.0589, 1.0899)	14.7%
% males	31	-0.17058	(-0.7450, 0.40959)	0%
Publication year	43	0.08228	(-0.5169, 0.71206)	0%
Hypertension	31	-0.07824	(-0.6629, 0.4874)	0%
CAD	16	0.20928	(-0.9908, 0.5744)	30.7%
SHD	43	-0.13467	(-0.6311, 0.36024)	0%
Left atrial dimension	35	0.27652	(-0.40740, 0.968495)	0%
Duration of follow-up	43	0.15622	(-0.2659, 0.60456)	0%
AF detection device	43	0.02865	(-0.5849, 0.53546)	0%

Section S12. SENSITIVITY ANALYSES

1 Excluding high risk of bias RCTs (38 RCTs left)

τ^2= 0.06253

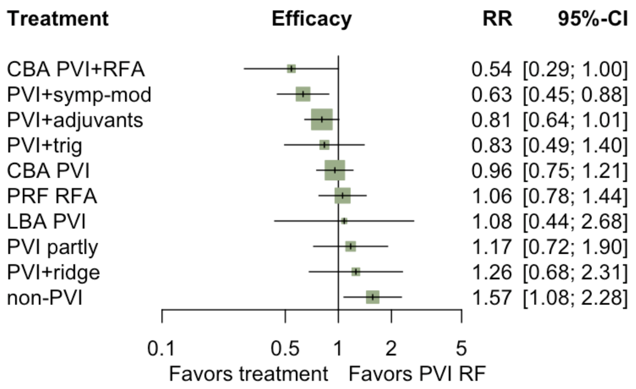

τ^2=0

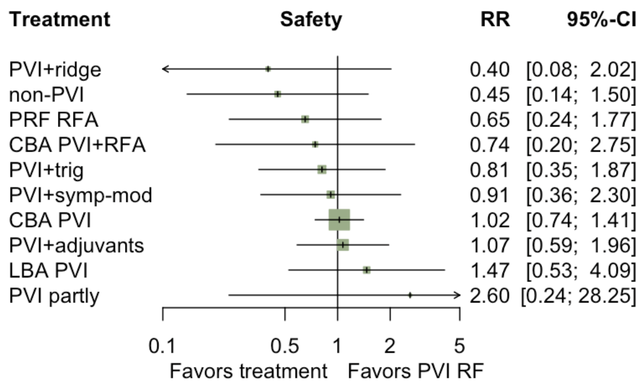

2 Excluding catheter 8mm, 8mm plus 3.5mm irrigated, 8mm and 4mm irrigated (39 RCTs left)

τ^2=0.0546

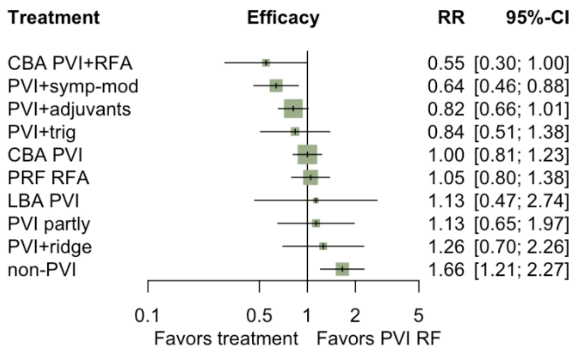

τ^2=0

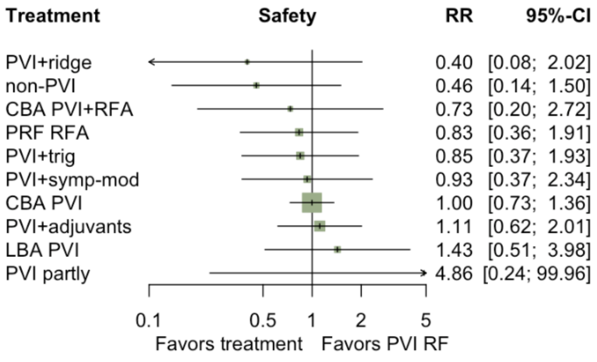

3 INCLUDING RCTs with antiarrhythmic drugs (AAD) (46 total RCTs)

Table S9. Characteristics of the additional 11 RCTs included in sensitivity network meta-analysis, including also RCTs with AADs as comparison arm.

Study	Year of publication	blanking period (weeks)	follow up period (months)	total number of patients	Strategies	Number of patients	Age (mean ± SD)	Sex (% male)	Hypertension (%)	CAD (%)	CHF (%)	SHD (%)	LVEF % (mean±SD)	LAD mm(mean±SD)
Pappone et al.(82)	2006	6	12	198	PVI+adjuvant	99	55±10	69 (70)	55 (56)	2 (2)	NR	NR	60±8	40±6
					AADs	99	57±10	64 (65)	56 (57)	2 (2)	NR	NR	61±6	38±6
Sohara et al.(83)	2016	12	9	153	HBA PVI	100	59±10	80 (80)	51 (51)	3 (3)	NR	NR	67±6	38±6
					AADs	43	61±10	35 (81)	24 (56)	2 (5)	NR	NR	67±7	38±5
Cosedis Nielsen et al.(48)	2012	12	24		PVI+adjuvant	146	56±9	100 (68)	43 (29)	6 (4)	15 (10)	NR	NR	40±6
					AADs	148	54±10	106 (72)	53 (36)	2 (1)	20 (14)	NR	NR	40±5

table S10 Risk of Bias assessment with domains (additional 3 RCTs of the AADs sensitivity analysis).

Risk of bias domains

Study	D1	D2	D3	D4	D5	Overall
Cosedis Nielsen et al.	+	X	+	+	+	X
Pappone et al.	-	X	+	+	+	X
Sohara et al.	-	X	+	+	+	X

Domains:
D1: Bias arising from the randomization process.
D2: Bias due to deviations from intended intervention.
D3: Bias due to missing outcome data.
D4: Bias in measurement of the outcome.
D5: Bias in selection of the reported result.

Judgement
X High
- Some concerns
+ Low

Figure S8: Network forest plots and network graphs for efficacy and safety vs PVI (including AADs as control arm)

τ^2=0.07

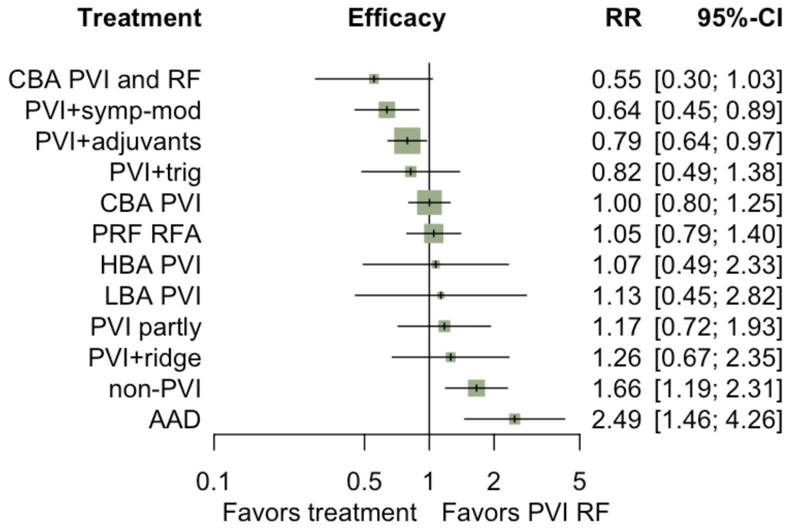

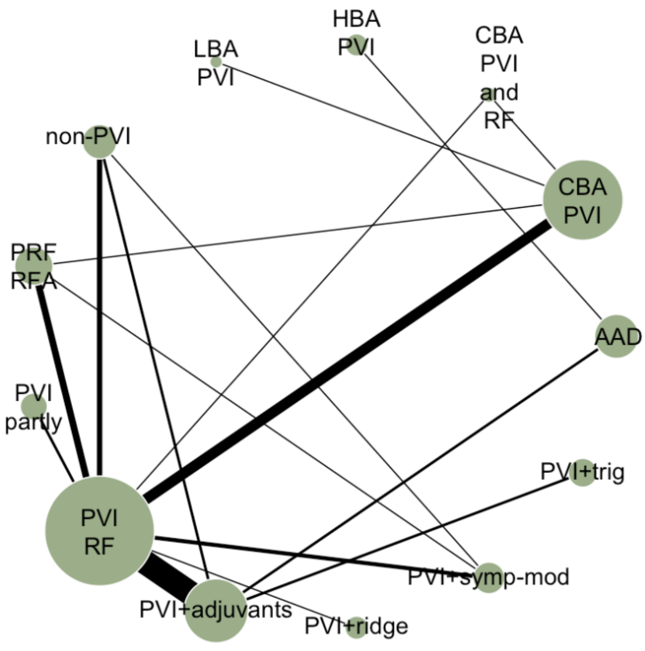

τ^2=0.13

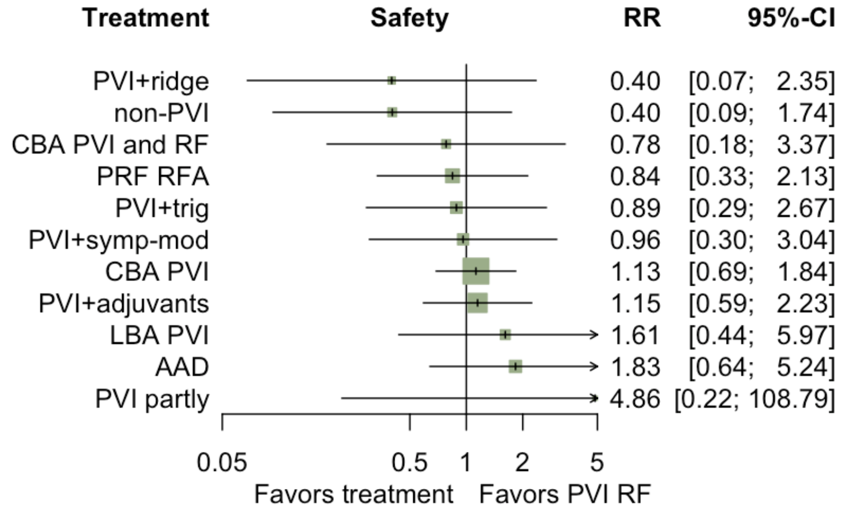
Treatment
Safety
RR
95%-CI
PVI+ridge 0.40 [0.07; 2.35]
non-PVI 0.40 [0.09; 1.74]
CBA PVI and RF 0.78 [0.18; 3.37]
PRF RFA 0.84 [0.33; 2.13]
PVI+trig 0.89 [0.29; 2.67]
PVI+symp-mod 0.96 [0.30; 3.04]
CBA PVI 1.13 [0.69; 1.84]
PVI+adjuvants 1.15 [0.59; 2.23]
LBA PVI 1.61 [0.44; 5.97]
AAD 1.83 [0.64; 5.24]
PVI partly 4.86 [0.22; 108.79]
0.05
0.5
1
2
5
Favors treatment
Favors PVI RF

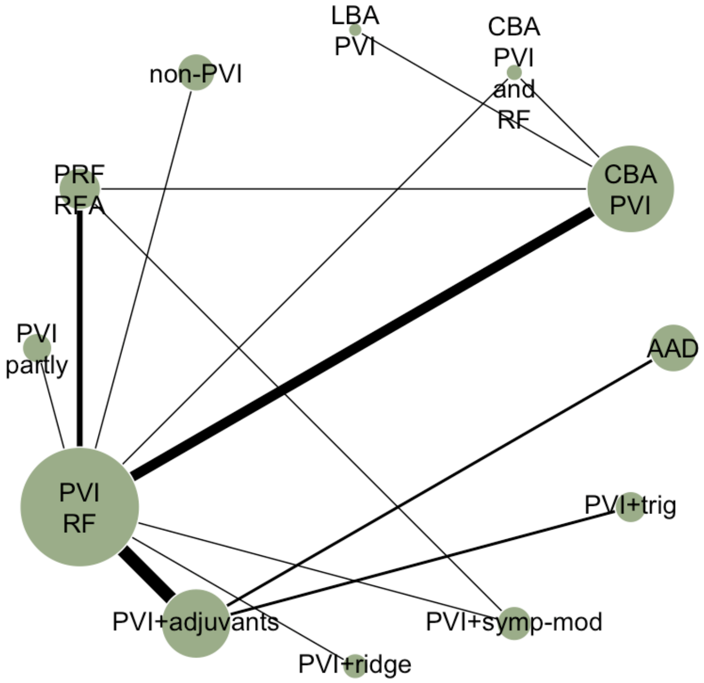
LBA PVI
CBA PVI and RF
non-PVI
PRF RFA
CBA PVI
PVI partly
AAD
PVI RF
PVI+trig
PVI+adjuvants
PVI+symp-mod
PVI+ridge

Figure S9: NMA forest plots for efficacy and safety vs AAD

(a)

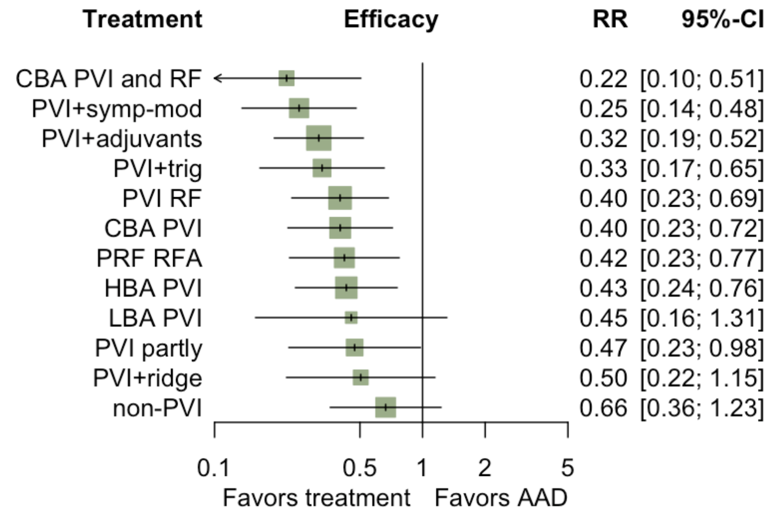

(b)

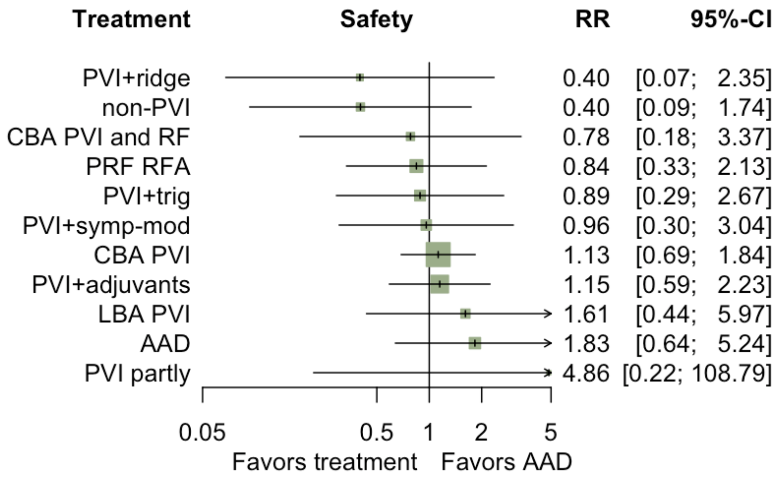

Section S13. OVERALL QUALITY OF EVIDENCE WITH CINeMA ASSESSMENT

Confidence in Network Meta-Analysis (CINeMA) tool (https://cinema.ispm. unibe.ch):

CINeMA is an online tool which allows for an automated evaluation of the quality of evidence of every comparison in the network. CINeMA considers six domains that may affect the level of confidence in the NMA results at any stage of the systematic review: (1) within-study bias, (2) reporting bias, (3) indirectness, (4) imprecision, (5) heterogeneity, and (6) incoherence. For each relative treatment effect from NMA reviewers pick "no concerns," "some concerns," or "major concerns" in each of the six domains, which are then summarized into four confidence domains ("high," "moderate," "low," or "very low").

Specifically, the confidence in each NMA risk ratio, RR_{XY} (with two given interventions X and Y) was evaluated for all the six domains. For each comparison and for each domain, the user is required to input his judgement to recommend whether there were 'major concerns', 'some concerns' or 'no concerns' for the particular comparison and domain. Thresholds and evaluation rules are typically decided through discussions.

For indirectness the judgements take into account the relative contributions of direct and indirect studies in the estimation. For imprecision, heterogeneity and incoherence, the tool uses the concept of the minimally clinically important effect size and constructs the 'range of equivalence' between two interventions. Then, judgements about the three domains consider whether uncertainty intervals lie within this range.

1. Within-trial bias: was estimated as the weighted average of the overall risk of bias of all the trials contributing information to the estimation of RRs.
2. Reporting bias (or 'publication bias'): was set it to 'low risk' for all studies, as small-study effects assessment did not suggest the presence of publication bias and the risk of publication bias based on the knowledge of the clinical field did not raise any concerns.
3. Indirectness: Indirectness was summarized in each comparison as average for each relative effect estimate according to the percentage contribution of studies at each bias level.
4. Imprecision: we left the clinically important size of the effect (RR) to 1 as we had no clinical reason to assume otherwise.
5. Heterogeneity: was evaluated by monitoring agreement between confidence intervals (CIs) and prediction intervals (PIs) in terms of their ability to provide similar conclusions.
6. Incoherence: was evaluated by monitoring disagreement between confidence intervals (CIs) of the direct and indirect RR_{XY} and their overlap with the margin of equivalent effects.

In both networks, reasons for downgrading to moderate or low certainty were decided based on the presence of high concerns in imprecision, either alone or accompanied by some concerns in incoherence or indirectness, respectively. In this analysis, within-study bias was expected to have

some concerns of bias as blinding of participants was not possible, thus this domain was not considered to further down-grade evidence alone.

We report the results of evaluation of certainty of evidence for the primary efficacy and safety outcomes in eTable 8 and eTable 9, as either no concern, some concerns or major concerns for each domain assessed (within-study bias, reporting bias, indirectness, imprecision, heterogeneity and incoherence). For efficacy, we also report the contribution of medium or high risk of bias (RoB) comparisons to each network estimate and the contribution of indirectness.

Reasons for downgrading the evidence were related to the presence of concerns imprecision, within-study bias, indirectness and/or heterogeneity. However, the domains can often be considered jointly. For example, if some concerns are present in both indirectness and incoherence, we considered sufficient to downgrade the evidence only by one level. This is because indirectness and incoherence are known to be related one another. Assessment of indirectness of the research question include considerations about transitivity, but on the other hand, transitivity concerns appear in the data in the form of statistical incoherence. Similarly, high heterogeneity increases imprecision so these two domains are also interconnected. Evidence was downgraded by one level if "some concerns" were present in two or more domains, whilst the presence of "major concerns" in a domain was considered enough to downgrade alone the evidence by one level. The two rules apply cumulatively and when jointly occurring can downgrade evidence by two or more levels.

Final assessment for Efficacy:

Comparison	Number of studies	Within-study bias	Reporting bias	Indirectness	Imprecision	Heterogeneity	Incoherence	Confidence rating	Reason(s) for downgrading
**CBA PVI:CBA PVI+RFA**	1	Some concerns	Low risk	No concerns	Major concerns	No concerns	No concerns	Moderate	["Imprecision"]
**CBA PVI:LBA PVI**	1	Some concerns	Low risk	No concerns	Major concerns	No concerns	Some concerns	Low	[“Within-study bias”, "Imprecision”/"Incoherence"]
**CBA PVI:PRF RFA**	1	Some concerns	Low risk	No concerns	Major concerns	No concerns	No concerns	Moderate	["Imprecision"]
**CBA PVI:PVI RF**	8	Some concerns	Low risk	No concerns	Major concerns	No concerns	No concerns	Moderate	["Imprecision"]
**CBA PVI+RFA:PVI RF**	1	Some concerns	Low risk	No concerns	Major concerns	No concerns	No concerns	Moderate	["Imprecision"]
**PRF RFA:PVI RF**	4	Some concerns	Low risk	No concerns	Major concerns	No concerns	No concerns	Moderate	["Imprecision"]
**PRF RFA:PVI+symp-mod**	1	Some concerns	Low risk	No concerns	No concerns	Major concerns	No concerns	Moderate	["Heterogeneity"]
**PVI partly:PVI RF**	2	Some concerns	Low risk	No concerns	Major concerns	No concerns	Some concerns	Low	[“Within-study bias”, "Imprecision”/"Incoherence"]
**PVI+adjuvants:PVI RF**	18	Some concerns	Low risk	No concerns	No concerns	Major concerns	No concerns	Moderate	["Heterogeneity"]
**PVI RF:PVI+ridge**	1	Some concerns	Low risk	No concerns	Major concerns	No concerns	Some concerns	Low	[“Within-study bias”, "Imprecision”/"Incoherence"]
**PVI RF:PVI+symp-mod**	3	Some concerns	Low risk	Some concerns	No concerns	Major concerns	No concerns	Low	[“Within-study bias”, "Heterogeneity "]
**non-PVI:PVI RF**	4	Some concerns	Low risk	No concerns	No concerns	Major concerns	No concerns	Moderate	["Heterogeneity"]
**PVI+adjuvants:PVI+trig**	2	Some concerns	Low risk	Some concerns	Major concerns	No concerns	Some concerns	Low	[“Within-study bias”, "Imprecision"]
**non-PVI:PVI+adjuvants**	2	Some concerns	Low risk	No concerns	No concerns	No concerns	No concerns	High	[]
**non-PVI:PVI+symp-mod**	1	Some concerns	Low risk	No concerns	No concerns	No concerns	No concerns	High	[]
**CBA PVI:PVI partly**	0	Some concerns	Low risk	No concerns	Major concerns	No concerns	Some concerns	Low	[“Within-study bias”, "Imprecision"]
**CBA PVI:PVI+adjuvants**	0	Some concerns	Low risk	No concerns	Major concerns	No concerns	Some concerns	Low	[“Within-study bias”, "Imprecision"]
**CBA PVI:PVI+ridge**	0	Some concerns	Low risk	No concerns	Major concerns	No concerns	Some concerns	Low	[“Within-study bias”, "Imprecision"]

**CBA PVI:PVI+symp-mod**	0	Some concerns	Low risk	No concerns	No concerns	Major concerns	Some concerns	Low	[“Within-study bias”, "Imprecision"]
**CBA PVI:PVI+trig**	0	Some concerns	Low risk	No concerns	Major concerns	No concerns	Some concerns	Low	[“Within-study bias”, "Imprecision"]
**CBA PVI:non-PVI**	0	Some concerns	Low risk	No concerns	No concerns	Major concerns	Some concerns	Low	[“Within-study bias”, "Imprecision"]
**CBA PVI+RFA:LBA PVI**	0	Some concerns	Low risk	No concerns	Major concerns	No concerns	Some concerns	Low	[“Within-study bias”, "Imprecision"]
**CBA PVI+RFA:PRF RFA**	0	Some concerns	Low risk	No concerns	Major concerns	No concerns	Some concerns	Low	[“Within-study bias”, "Imprecision"]
**CBA PVI+RFA:PVI partly**	0	Some concerns	Low risk	No concerns	Major concerns	No concerns	Some concerns	Low	[“Within-study bias”, "Imprecision"]
**CBA PVI+RFA:PVI+adjuvants**	0	Some concerns	Low risk	No concerns	Major concerns	No concerns	Some concerns	Low	[“Within-study bias”, "Imprecision"]
**CBA PVI+RFA:PVI+ridge**	0	Some concerns	Low risk	No concerns	Major concerns	No concerns	Some concerns	Low	[“Within-study bias”, "Imprecision"]
**CBA PVI+RFA:PVI+symp-mod**	0	Some concerns	Low risk	No concerns	Major concerns	No concerns	Some concerns	Low	[“Within-study bias”, "Imprecision"]
**CBA PVI+RFA:PVI+trig**	0	Some concerns	Low risk	No concerns	Major concerns	No concerns	Some concerns	Low	[“Within-study bias”, "Imprecision"]
**CBA PVI+RFA:non-PVI**	0	Some concerns	Low risk	No concerns	No concerns	No concerns	Some concerns	Moderate	[“Within-study bias”/”Incoherence”]
**LBA PVI:PRF RFA**	0	Some concerns	Low risk	No concerns	Major concerns	No concerns	Some concerns	Low	[“Within-study bias”, "Imprecision"]
**LBA PVI:PVI RF**	0	Some concerns	Low risk	No concerns	Major concerns	No concerns	Some concerns	Low	[“Within-study bias”, "Imprecision"]
**LBA PVI:PVI partly**	0	Some concerns	Low risk	No concerns	Major concerns	No concerns	Some concerns	Low	[“Within-study bias”, "Imprecision"]
**LBA PVI:PVI+adjuvants**	0	Some concerns	Low risk	No concerns	Major concerns	No concerns	Some concerns	Low	[“Within-study bias”, "Imprecision"]
**LBA PVI:PVI+ridge**	0	Some concerns	Low risk	No concerns	Major concerns	No concerns	Some concerns	Low	[“Within-study bias”, "Imprecision"]
**LBA PVI:PVI+symp-mod**	0	Some concerns	Low risk	No concerns	Major concerns	No concerns	Some concerns	Low	[“Within-study bias”, "Imprecision"]
**LBA PVI:PVI+trig**	0	Some concerns	Low risk	No concerns	Major concerns	No concerns	Some concerns	Low	[“Within-study bias”, "Imprecision"]
**LBA PVI:non-PVI**	0	Some concerns	Low risk	No concerns	Major concerns	No concerns	Some concerns	Low	[“Within-study bias”, "Imprecision"]
**PRF RFA:PVI partly**	0	Some concerns	Low risk	No concerns	Major concerns	No concerns	Some concerns	Low	[“Within-study bias”, "Imprecision"]

**PRF RFA:PVI+adjuvants**	0	Some concerns	Low risk	No concerns	Major concerns	No concerns	Some concerns	Low	[“Within-study bias”, "Imprecision"]
**PRF RFA:PVI+ridge**	0	Some concerns	Low risk	No concerns	Major concerns	No concerns	Some concerns	Low	[“Within-study bias”, "Imprecision"]
**PRF RFA:PVI+trig**	0	Some concerns	Low risk	No concerns	Major concerns	No concerns	Some concerns	Low	[“Within-study bias”, "Imprecision"]
**non-PVI:PRF RFA**	0	Some concerns	Low risk	No concerns	No concerns	Major concerns	Some concerns	Low	[“Within-study bias”, "Heterogeneity"]
**PVI RF:PVI+trig**	0	Some concerns	Low risk	Some concerns	Major concerns	No concerns	Some concerns	Low	[“Within-study bias”, "Imprecision"]
**PVI+adjuvants:PVI partly**	0	Some concerns	Low risk	No concerns	Major concerns	No concerns	Some concerns	Low	[“Within-study bias”, "Imprecision"]
**PVI partly:PVI+ridge**	0	Some concerns	Low risk	No concerns	Major concerns	No concerns	Some concerns	Low	[“Within-study bias”, "Imprecision"]
**PVI partly:PVI+symp-mod**	0	Some concerns	Low risk	No concerns	No concerns	Major concerns	Some concerns	Low	[“Within-study bias”, "Heterogeneity"]
**PVI partly:PVI+trig**	0	Some concerns	Low risk	No concerns	Major concerns	No concerns	Some concerns	Low	[“Within-study bias”, "Imprecision"]
**non-PVI:PVI partly**	0	Some concerns	Low risk	No concerns	Major concerns	No concerns	Some concerns	Low	[“Within-study bias”, "Imprecision"]
**PVI+adjuvants:PVI+ridge**	0	Some concerns	Low risk	No concerns	Major concerns	No concerns	Some concerns	Low	[“Within-study bias”, "Imprecision"]
**PVI+adjuvants:PVI+symp-mod**	0	Some concerns	Low risk	No concerns	Major concerns	No concerns	Some concerns	Low	[“Within-study bias”, "Imprecision"]
**PVI+ridge:PVI+symp-mod**	0	Some concerns	Low risk	No concerns	No concerns	Major concerns	Some concerns	Low	[“Within-study bias”, "Heterogeneity"]
**PVI+ridge:PVI+trig**	0	Some concerns	Low risk	No concerns	Major concerns	No concerns	Some concerns	Low	[“Within-study bias”, "Imprecision"]
**non-PVI:PVI+ridge**	0	Some concerns	Low risk	No concerns	Major concerns	No concerns	Some concerns	Low	[“Within-study bias”, "Imprecision"]
**PVI+symp-mod:PVI+trig**	0	Some concerns	Low risk	Some concerns	Major concerns	No concerns	Some concerns	Low	[“Within-study bias”, "Imprecision"]
**non-PVI:PVI+trig**	0	Some concerns	Low risk	No concerns	No concerns	Major concerns	Some concerns	Low	[“Within-study bias”, "Heterogeneity"]

Final assessment for Safety:

Comparison	Number of studies	Within-study bias	Reporting bias	Indirectness	Imprecision	Heterogeneity	Incoherence	Confidence rating	Reason(s) for downgrading
**CBA PVI:CBA PVI+RFA**	1	Some concerns	Low risk	No concerns	Major concerns	No concerns	No concerns	Moderate	["Imprecision"]
**CBA PVI:LBA PVI**	1	Some concerns	Low risk	No concerns	Major concerns	No concerns	No concerns	Moderate	["Imprecision"]
**CBA PVI:PRF RFA**	1	Some concerns	Low risk	No concerns	Major concerns	No concerns	No concerns	Moderate	["Imprecision"]
**CBA PVI:PVI RF**	7	Some concerns	Low risk	No concerns	Major concerns	No concerns	No concerns	Moderate	["Imprecision"]
**CBA PVI+RFA:PVI RF**	1	Some concerns	Low risk	No concerns	Major concerns	No concerns	No concerns	Moderate	["Imprecision"]
**PRF RFA:PVI RF**	4	Some concerns	Low risk	No concerns	Major concerns	No concerns	No concerns	Moderate	["Imprecision"]
**PRF RFA:PVI+symp-mod**	1	Some concerns	Low risk	No concerns	Major concerns	No concerns	No concerns	Moderate	["Imprecision"]
**PVI partly:PVI RF**	2	Some concerns	Low risk	No concerns	Major concerns	No concerns	No concerns	Moderate	["Imprecision"]
**PVI+adjuvants:PVI RF**	12	Some concerns	Low risk	No concerns	Major concerns	No concerns	No concerns	Moderate	["Imprecision"]
**PVI RF:PVI+ridge**	1	Some concerns	Low risk	No concerns	Major concerns	No concerns	No concerns	Moderate	["Imprecision"]
**PVI RF:PVI+symp-mod**	3	Some concerns	Low risk	Some concerns	Major concerns	No concerns	No concerns	Low	["Within study bias","Imprecision"]
**non-PVI:PVI RF**	3	Some concerns	Low risk	No concerns	Major concerns	No concerns	No concerns	Moderate	["Imprecision"]
**PVI+adjuvants:PVI+trig**	2	Some concerns	Low risk	Some concerns	Major concerns	No concerns	No concerns	Low	["Within study bias","Imprecision"]
**non-PVI:PVI+adjuvants**	1	Some concerns	Low risk	No concerns	Major concerns	No concerns	No concerns	Moderate	["Imprecision"]
**non-PVI:PVI+symp-mod**	1	Some concerns	Low risk	No concerns	Major concerns	No concerns	No concerns	Moderate	["Imprecision"]
**CBA PVI:PVI partly**	0	Some concerns	Low risk	No concerns	Major concerns	No concerns	No concerns	Moderate	["Imprecision"]
**CBA PVI:PVI+adjuvants**	0	Some concerns	Low risk	No concerns	Major concerns	No concerns	No concerns	Moderate	["Imprecision"]
**CBA PVI:PVI+ridge**	0	Some concerns	Low risk	No concerns	Major concerns	No concerns	No concerns	Moderate	["Imprecision"]

**CBA PVI:PVI+symp-mod**	0	Some concerns	Low risk	No concerns	Major concerns	No concerns	No concerns	Moderate	["Imprecision"]
**CBA PVI:PVI+trig**	0	Some concerns	Low risk	No concerns	Major concerns	No concerns	No concerns	Moderate	["Imprecision"]
**CBA PVI:non-PVI**	0	Some concerns	Low risk	No concerns	Major concerns	No concerns	No concerns	Moderate	["Imprecision"]
**CBA PVI+RFA:LBA PVI**	0	Some concerns	Low risk	No concerns	Major concerns	No concerns	No concerns	Moderate	["Imprecision"]
**CBA PVI+RFA:PRF RFA**	0	Some concerns	Low risk	No concerns	Major concerns	No concerns	No concerns	Moderate	["Imprecision"]
**CBA PVI+RFA:PVI partly**	0	Some concerns	Low risk	No concerns	Major concerns	No concerns	No concerns	Moderate	["Imprecision"]
**CBA PVI+RFA:PVI+adjuvants**	0	Some concerns	Low risk	No concerns	Major concerns	No concerns	No concerns	Moderate	["Imprecision"]
**CBA PVI+RFA:PVI+ridge**	0	Some concerns	Low risk	No concerns	Major concerns	No concerns	No concerns	Moderate	["Imprecision"]
**CBA PVI+RFA:PVI+symp-mod**	0	Some concerns	Low risk	No concerns	Major concerns	No concerns	No concerns	Moderate	["Imprecision"]
**CBA PVI+RFA:PVI+trig**	0	Some concerns	Low risk	No concerns	Major concerns	No concerns	No concerns	Moderate	["Imprecision"]
**CBA PVI+RFA:non-PVI**	0	Some concerns	Low risk	No concerns	Major concerns	No concerns	No concerns	Moderate	["Imprecision"]
**LBA PVI:PRF RFA**	0	Some concerns	Low risk	No concerns	Major concerns	No concerns	No concerns	Moderate	["Imprecision"]
**LBA PVI:PVI RF**	0	Some concerns	Low risk	No concerns	Major concerns	No concerns	No concerns	Moderate	["Imprecision"]
**LBA PVI:PVI partly**	0	Some concerns	Low risk	No concerns	Major concerns	No concerns	No concerns	Moderate	["Imprecision"]
**LBA PVI:PVI+adjuvants**	0	Some concerns	Low risk	No concerns	Major concerns	No concerns	No concerns	Moderate	["Imprecision"]
**LBA PVI:PVI+ridge**	0	Some concerns	Low risk	No concerns	Major concerns	No concerns	No concerns	Moderate	["Imprecision"]
**LBA PVI:PVI+symp-mod**	0	Some concerns	Low risk	No concerns	Major concerns	No concerns	No concerns	Moderate	["Imprecision"]
**LBA PVI:PVI+trig**	0	Some concerns	Low risk	No concerns	Major concerns	No concerns	No concerns	Moderate	["Imprecision"]
**LBA PVI:non-PVI**	0	Some concerns	Low risk	No concerns	Major concerns	No concerns	No concerns	Moderate	["Imprecision"]
**PRF RFA:PVI partly**	0	Some concerns	Low risk	No concerns	Major concerns	No concerns	No concerns	Moderate	["Imprecision"]

**PRF RFA:PVI+adjuvants**	0	Some concerns	Low risk	No concerns	Major concerns	No concerns	No concerns	Moderate	["Imprecision"]
**PRF RFA:PVI+ridge**	0	Some concerns	Low risk	No concerns	Major concerns	No concerns	No concerns	Moderate	["Imprecision"]
**PRF RFA:PVI+trig**	0	Some concerns	Low risk	No concerns	Major concerns	No concerns	No concerns	Moderate	["Imprecision"]
**non-PVI:PRF RFA**	0	Some concerns	Low risk	No concerns	Major concerns	No concerns	No concerns	Moderate	["Imprecision"]
**PVI RF:PVI+trig**	0	Some concerns	Low risk	Some concerns	Major concerns	No concerns	No concerns	Low	["Within study bias","Imprecision"]
**PVI+adjuvants:PVI partly**	0	Some concerns	Low risk	No concerns	Major concerns	No concerns	No concerns	Moderate	["Imprecision"]
**PVI partly:PVI+ridge**	0	Some concerns	Low risk	No concerns	Major concerns	No concerns	No concerns	Moderate	["Imprecision"]
**PVI partly:PVI+symp-mod**	0	Some concerns	Low risk	No concerns	Major concerns	No concerns	No concerns	Moderate	["Imprecision"]
**PVI partly:PVI+trig**	0	Some concerns	Low risk	No concerns	Major concerns	No concerns	No concerns	Moderate	["Imprecision"]
**non-PVI:PVI partly**	0	Some concerns	Low risk	No concerns	Major concerns	No concerns	No concerns	Moderate	["Imprecision"]
**PVI+adjuvants:PVI+ridge**	0	Some concerns	Low risk	No concerns	Major concerns	No concerns	No concerns	Moderate	["Imprecision"]
**PVI+adjuvants:PVI+symp-mod**	0	Some concerns	Low risk	Some concerns	Major concerns	No concerns	No concerns	Low	["Within study bias","Imprecision"]
**PVI+ridge:PVI+symp-mod**	0	Some concerns	Low risk	No concerns	Major concerns	No concerns	No concerns	Moderate	["Imprecision"]
**PVI+ridge:PVI+trig**	0	Some concerns	Low risk	No concerns	Major concerns	No concerns	No concerns	Moderate	["Imprecision"]
**non-PVI:PVI+ridge**	0	Some concerns	Low risk	No concerns	Major concerns	No concerns	No concerns	Moderate	["Imprecision"]
**PVI+symp-mod:PVI+trig**	0	Some concerns	Low risk	Some concerns	Major concerns	No concerns	No concerns	Low	["Within study bias","Imprecision"]
**non-PVI:PVI+trig**	0	Some concerns	Low risk	Some concerns	Major concerns	No concerns	No concerns	Low	["Within study bias","Imprecision"]

References

All the references cited in the supplement are presented with their reference number on the reference list of the main manuscript.
